# *CHD8* suppression impacts on histone H3 lysine 36 trimethylation and alters RNA alternative splicing

**DOI:** 10.1093/nar/gkac1134

**Published:** 2022-12-20

**Authors:** Emanuela Kerschbamer, Michele Arnoldi, Takshashila Tripathi, Miguel Pellegrini, Samuele Maturi, Serkan Erdin, Elisa Salviato, Francesca Di Leva, Endre Sebestyén, Erik Dassi, Giulia Zarantonello, Matteo Benelli, Eric Campos, M Albert Basson, James F Gusella, Stefano Gustincich, Silvano Piazza, Francesca Demichelis, Michael E Talkowski, Francesco Ferrari, Marta Biagioli

**Affiliations:** NeuroEpigenetics laboratory, Department of Cellular, Computational and Integrative Biology, (CIBIO) University of Trento, Trento, Italy; NeuroEpigenetics laboratory, Department of Cellular, Computational and Integrative Biology, (CIBIO) University of Trento, Trento, Italy; NeuroEpigenetics laboratory, Department of Cellular, Computational and Integrative Biology, (CIBIO) University of Trento, Trento, Italy; NeuroEpigenetics laboratory, Department of Cellular, Computational and Integrative Biology, (CIBIO) University of Trento, Trento, Italy; NeuroEpigenetics laboratory, Department of Cellular, Computational and Integrative Biology, (CIBIO) University of Trento, Trento, Italy; Center for Genomic Medicine, Massachusetts General Hospital, Boston, MA, USA; Program in Medical and Population Genetics, Broad Institute, Cambridge, MA, USA; IFOM, the FIRC Institute of Molecular Oncology, Milan, Italy; NeuroEpigenetics laboratory, Department of Cellular, Computational and Integrative Biology, (CIBIO) University of Trento, Trento, Italy; IFOM, the FIRC Institute of Molecular Oncology, Milan, Italy; Laboratory of RNA Regulatory Networks, Department of Cellular, Computational and Integrative Biology, (CIBIO), University of Trento, Trento, Italy; NeuroEpigenetics laboratory, Department of Cellular, Computational and Integrative Biology, (CIBIO) University of Trento, Trento, Italy; Bioinformatics Unit, Hospital of Prato, Istituto Toscano Tumori, Prato, Italy; Genetics & Genome Biology Program, The Hospital for Sick Children, Toronto, Canada; Department of Molecular Genetics, University of Toronto, Toronto, Canada; Centre for Craniofacial and Regenerative Biology and MRC Centre for Neurodevelopmental Disorders, King's College London, London, UK; Center for Genomic Medicine, Massachusetts General Hospital, Boston, MA, USA; Program in Medical and Population Genetics, Broad Institute, Cambridge, MA, USA; Department of Neurology, Harvard Medical School, Boston, MA, USA; Central RNA Laboratory, Istituto Italiano di Tecnologia (IIT), Genova, Italy; Bioinformatic facility, Department of Cellular, Computational and Integrative Biology (CIBIO) University of Trento, Italy; Laboratory of Computational and Functional Oncology, Department of Cellular, Computational and Integrative Biology (CIBIO), University of Trento, Trento, Italy; Center for Genomic Medicine, Massachusetts General Hospital, Boston, MA, USA; Program in Medical and Population Genetics, Broad Institute, Cambridge, MA, USA; Department of Neurology, Harvard Medical School, Boston, MA, USA; IFOM, the FIRC Institute of Molecular Oncology, Milan, Italy; CNR Institute of Molecular Genetics ‘Luigi Luca Cavalli-Sforza’, Pavia, Italy; NeuroEpigenetics laboratory, Department of Cellular, Computational and Integrative Biology, (CIBIO) University of Trento, Trento, Italy

## Abstract

Disruptive mutations in the chromodomain helicase DNA-binding protein 8 gene (*CHD8*) have been recurrently associated with autism spectrum disorders (ASDs). Here we investigated how chromatin reacts to *CHD8* suppression by analyzing a panel of histone modifications in induced pluripotent stem cell-derived neural progenitors. *CHD8* suppression led to significant reduction (47.82%) in histone H3K36me3 peaks at gene bodies, particularly impacting on transcriptional elongation chromatin states. H3K36me3 reduction specifically affects highly expressed, CHD8-bound genes and correlates with altered alternative splicing patterns of 462 genes implicated in ‘regulation of RNA splicing’ and ‘mRNA catabolic process’. Mass spectrometry analysis uncovered a novel interaction between CHD8 and the splicing regulator heterogeneous nuclear ribonucleoprotein L (hnRNPL), providing the first mechanistic insights to explain the *CHD8* suppression-derived splicing phenotype, partly implicating SETD2, a H3K36me3 methyltransferase. In summary, our results point toward broad molecular consequences of *CHD8* suppression, entailing altered histone deposition/maintenance and RNA processing regulation as important regulatory processes in ASD.

## INTRODUCTION

*De novo* truncating mutations in the chromodomain helicase DNA-binding protein 8 gene (*CHD8*) have been reported and independently validated to be a strong risk factor for autism spectrum disorders (ASDs) (1–7). *CHD8* has been classified as a high confidence ASD candidate risk factor (score 1) in the Simons Foundation Autism Research Initiative (SFARI) [https://gene.sfari.org (8)]. More than 50% of reported *CHD8* variants associated with the disease can be classified as putative loss-of-function mutations (copy number loss, frameshift variant, stop gained and translocation) (59.18% ClinVar database https://www.ncbi.nlm.nih.gov/clinvar/; 56.36% SFARI database https://gene.sfari.org/) and are more likely to result in protein haploinsufficiency. *CHD8* defines a subclass of ASD patients, displaying evident macrocephaly, distinct faces, sleep problems and gastrointestinal complaints (9,10). Most of these phenotypic characteristics were recapitulated in *chd8* knockdown zebrafish (9,11) and, more recently, in *Chd8* suppression mouse models (12–15). Indeed, *chd8*-morpholino zebrafish and *Chd8* heterozygous mice display increased brain size, possibly initiated by altered gene expression in the developing brain areas (11,14). Remarkably, genome-wide transcriptomic changes that impact on ASD-related genes were also detected *in vitro*, in human neural progenitor cells (hNPCs) with reduced *CHD8* expression (11). Taken together, these observations suggest that aberrant genome-wide transcription leading to altered brain development is strictly correlated to reduced levels of CHD8 function. However, the detailed molecular mechanism through which CHD8 regulates this process still remains obscure.

A direct effect can be proposed since CHD8 is able to bind DNA at promoters and enhancer regions in hNPCs, mouse midfetal brain and embryonic cortex (11,16). However, an indirect mechanism can also be postulated since other genes, not bound by CHD8, appear to be transcriptionally dysregulated following *CHD8* suppression (11). Chromatin structure is intimately related to transcription and gene expression (17). CHD8 was shown to co-purify with components of the MLL and CoREST, SWI/SNF and NuRD ATP-dependent remodeling complexes, supporting its possible role in transcriptional initiation (18). On the other hand, reduction of CHD1, another member of the CHD protein family, alters H3K4me3 and H3K36me3 patterns, suggesting its role in establishing/maintaining the boundaries of these mutually exclusive histone marks (19). The SETD2 and SETD5 histone H3, Lys36 methyltransferases normally associate with RNA polymerase II (RNAPII), and their activity results in increased H3K36me3 toward the 3′ end of active genes (20,21). Based on its placement on the phylogenetic tree and the presence of an ATPase domain (18), CHD8 is most probably acting as an ATP-dependent chromatin-remodeling factor; thus, similar to CHD1, CHD8 loss might cause increased nucleosome turnover and alterations in co-transcriptional processes, such as cryptic transcription within gene bodies and alternative splicing (AS) (22,23). In addition to the general splicing machinery, many RNA-binding proteins (RBPs), such as serine-rich proteins (SR-proteins) and heterogeneous nuclear ribonucleoproteins (hnRNPs), function as splicing enhancers and silencers (24–26). Broad chromatin conformation and transcriptional kinetics also play a role: chromatin relaxation accelerates RNAPII processing and generally correlates with alternative exon skipping; conversely, packed nucleosomes slow down RNAPII progression, causing pausing of transcription and the inclusion of non-constitutive weak exons (27–34). Aberrant splicing, in turn, might contribute to altered neuronal development and dysfunction (35–38). Thus, as experimental evidence is pointing to dysregulated chromatin regulation as a key feature in the pathogenesis of ASD, it is tempting to hypothesize that chromatin function of CHD proteins, and CHD8 in particular, might act to regulate RNA transcription, elongation and processing, thereby being responsible for the characteristic neurodevelopmental effects observed in ASD.

In order to dissect this mechanism, we characterized the consequences of *CHD8* suppression on the chromatin landscape, analyzing different histone modifications using chromatin immunoprecipitation and sequencing (ChIP-seq). Specifically, we interrogated histone marks characteristic of transcriptionally active (H3K4me2, H3K4me3, H3K27ac and H3K36me3) and repressed regions (H3K27me3) as well as active/poised enhancers (H3K4me1 and H3K27ac) in control induced pluripotent stem cell (iPSC)-derived neuronal progenitors and in previously characterized lines where an ∼50% reduction in *CHD8* was obtained by lentiviral delivery of short hairpin RNAs (shRNAs) (11). We uncovered alterations affecting the H3K36me3 histone mark in the body of highly transcribed genes, which do not primarily affect RNA transcription, but rather alter AS of genes implicated in ‘mRNA catabolic process’, ‘regulation of RNA splicing’ and ‘translation initiation’. Strikingly, by mass spectrometry (MS) analysis in human neuronal progenitors, we identified and validated a novel, direct protein–protein interaction between CHD8 and hnRNPL. This splicing regulator, reported to be part of the human KMT3a/SET2 complex required for H3 Lys36 trimethylation activity (39,40), represents a new link bridging chromatin to RNA processing regulation with possible crucial implications for ASD and other presently incurable brain disorders.

## MATERIALS AND METHODS

### Cellular model

The human iPSC-derived NPC line GM8330-8 (41) were kindly provided by the laboratory of Dr Stephen Haggarty (Massachusetts General Hospital and Harvard Medical School, Boston, MA, USA). Sh1-*CHD8*, Sh2-*CHD8*, Sh4-*CHD8* and Sh-*GFP* (green fluorescent protein) lines were previously generated by lentiviral delivery of shRNAs targeting *CHD8* and *GFP* coding sequence, respectively (11).

Cells were cultured on poly-l-ornithine hydrobromide (20 μg/ml, Sigma)/laminin (3 μg/ml, Life Technologies)-coated plates in hiNPC medium [70% v/v Dulbecco’s modified Eagle’s medium (DMEM; Life Technologies) completed with 30% v/v HAM F12 (Euroclone), 2% v/v B27 (Life Technologies), 1% v/v penicillin–streptomycin solution (Life Technologies) and 1% v/v l-glutamine (Corning) and supplemented with epidermal growth factor (EGF; 20 ng/ml, Sigma), basic fibroblast growth factor (bFGF; 20 ng/ml, R&D) and heparin (5 μg/ml, Sigma)]. Semi-confluent monolayers of hiNPCs were maintained in a 5% CO_2_, 37°C humidified incubator.

### Chromatin immunoprecipitation and sequencing (ChIP-seq)

ChIP was performed using the protocol described by (42), with minor modifications. Briefly, ∼25 × 10^6^ iPSC-derived NPCs, controls and Shs-*CHD8* were fixed with 1% formaldehyde and incubated for 10 min at room temperature with rotation. The cross-linking was quenched by adding 1.1 ml of 2.5 M glycine and incubation for 5 min at room temperature with rotation. The cells were pelleted at 1000 rpm, resuspended in ice-cold phosphate-buffered saline (PBS)/protease inhibitor (PI), spun for 5 min at 1000 rpm, washed with ice-cold PBS twice, harvested, pelleted and directly resuspended in 300 μl of lysis buffer/PI [50 mM Tris–HCl (pH 8.1), 1% sodium dodecylsulfate (SDS), 10 mM EDTA], kept on ice for 10 min rotating occasionally and vortexed vigorously for 15 s every 3 min. Sonication of the 200–700 bp smear of the samples was accomplished using a Bioruptor sonicator (Diagenode), for a total of 45 min of sonication at full power and sonication cycles of 30 s on/30 s off. Samples were centrifuged at max speed for 10 min at 4°C. Then, sheared chromatin was diluted 10-fold in ChIP dilution buffer [16.7 mM Tris–HCl (pH 8.1), 167 mM NaCl, 0.01% SDS, 1.1% Triton X-100, 1.2 mM EDTA], supplemented with PI. A 50 μl aliquot of sheared chromatin was removed and stored at 4°C as the control aliquot (INPUT). Each sample was incubated at 4°C overnight with antibodies (20 μg/ChIP) of interest. The following primary antibodies were used: H3K27me3 (07-449, Millipore), H3K4me3 (Ab8580, Abcam), H3K36me3 (Ab9050, Abcam), H3K4me2 (Ab7766, Abcam), H3K4me1 (Ab8895, Abcam) and H3K27ac (Ab4729, Abcam). Chromatin–antibody complexes were precipitated with Dynabeads Protein A beads (Invitrogen) and washed sequentially with low salt [20 mM Tris–HCl (pH 8.1), 150 mM NaCl, 0.1% SDS, 1% Triton X-100, 2 mM EDTA], high salt [20 mM Tris–HCl (pH 8.1), 500 mM NaCl, 0.1% SDS, 1% Triton X-100, 2 mM EDTA], LiCl [10 mM Tris–HCl (pH 8.1), 0.25 M LiCl, 1% NP-40, 1% sodium deoxycholate, 1 mM EDTA] and TE wash buffer [10 mM Tris–HCl (pH 8.0), 1 mM EDTA]. Immunoprecipitated chromatin and INPUT samples were then eluted in elution buffer [TE plus 1% SDS, 150 mM NaCl, 5 mM dithiothreitol (DTT)], de-crosslinked at 65°C overnight and treated with proteinase K. DNA isolation was performed using phenol:chloroform:isoamyl alcohol. DNA was precipitated with 200 mM NaCl, supplemented with 30 μg of glycogen, washed with ethanol and then treated with RNase I (Invitrogen). Finally, DNA was purified with the MinElute Kit (Qiagen). Quantification of ChIP and INPUT DNA was accomplished using the Qubit 2.0 Fluorometer system (Invitrogen). ChIP-seq libraries were prepared starting from 5 ng of fragmented DNA using the NEBNext UltraII DNA Library preparation kit (Illumina) following the manufacturer's instructions with no modifications. In order to obtain enough material for sequencing, eight cycles of polymerase chain reaction (PCR) amplification were performed on adaptor-ligated fragments.

### Analysis of histone marks by ChIP-seq

**Figure 1.**
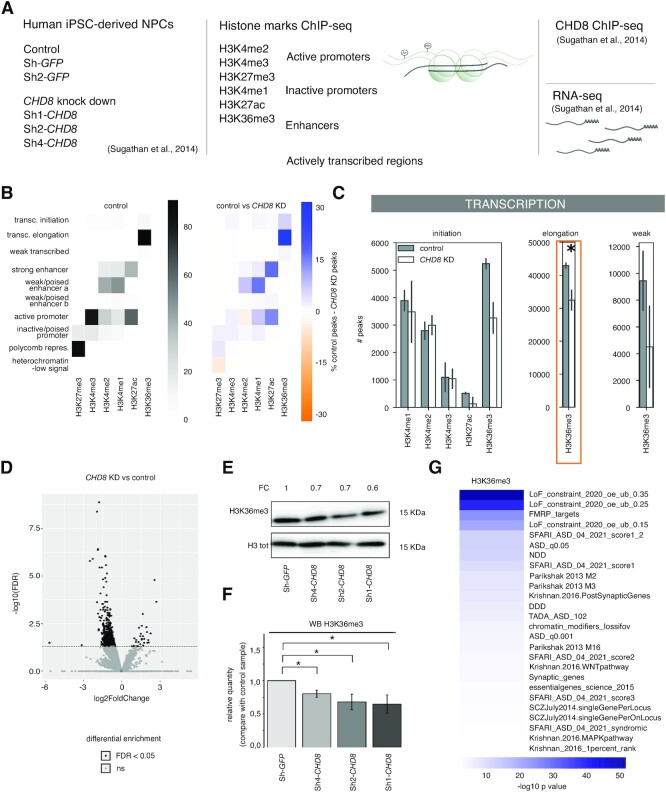
*CHD8* suppression significantly impacts on histone H3K36me3 enrichment at transcriptional elongation sites. (**A**) Schematic representation of the study design and integrative approach used in this work. Human iPSC-derived NPCs (hiNPC) knocked down for *CHD8* (Sh1-, Sh2- and Sh4-*CHD8*) and control hiNPCs (Sh-*GFP* and Sh-*GFP2*) (11), were analyzed via ChIP-seq for six histone marks representative of different chromatin regions: active promoters (H3K4me2 and H3K4me3), inactive promoters (H3K27me3), enhancers (H3K4me1 and H3K27ac) and actively transcribed regions (H3K36me3). ChIP-seq results were subsequently integrated with CHD8-binding sites and available transcriptomics (RNA-seq) datasets obtained from the same model system (11). (**B**) The heatmaps represent 10 different chromatin states (1, transcriptional initiation; 2, transcriptional elongation; 3, weakly transcribed; 4, strong enhancer; 5, weak/poised enhancer a; 6, weak/poised enhancer b; 7, active promoter; 8, inactive/poised promoter; 9, polycomb repressed; 10, heterochromatin/low signal), determined by the combination of different histone marks in control hiNPCs as defined by ChromHMM (48). The distribution of histone mark peaks across different chromatin states (see the Materials and Methods for details) is presented as a percentage of the total, and is color-coded in the heatmap (left). On the right, the heatmap describes the difference in number of peaks between two experimental conditions (controls versus *CHD8* knockdown). Chromatin states enriched in the control are indicated in blue and chromatin states enriched in *CHD8* knockdown in orange. H3K36me3 in transcriptional elongation is identified as the most affected chromatin state. (**C**) The bar plots represent the number of peaks for each histone mark identified at transcriptional initiation (left), elongation (center) and weakly transcribed (right) genomic regions. Gray bars indicate controls (*n* = 2, Sh-*GFP* and Sh-*GFP2*) and white bars refer to *CHD8* knockdown (*n* = 2, Sh2-*CHD8* and Sh4-*CHD8*). H3K36me3 peak loss upon *CHD8* suppression was significant at the transcriptional elongation states (two biological replicates, *t*-test, *P* <0.05). (**D**) The volcano plot reports differentially enriched peaks for H3K36me3 as detected by DiffBind (50,51). Peaks significantly different (FDR <0.05) are shown in black. Peaks not significantly different (ns, FDR >0.05) are shown in gray. The dashed horizontal line represents FDR = 0.05. Peaks enriched in *CHD8* knockdown compared with controls are represented on the right side of the plot, with positive log2(FC). Peaks depleted in *CHD8* knockdown (enriched in controls) are represented on the left side of the plot, with negative log2(FC). (**E**) A representative image illustrating total histone levels (H3K36me3 and H3 total), comparing control (Sh-*GFP*) and *CHD8* knockdown clones (Sh1-*CHD8*, Sh2-*CHD8* and Sh4-*CHD8*) from western blotting experiments. Levels of H3K36me3 reduction are indicated as FC compared with control Sh-*GFP*. Comparable amounts of total protein were loaded. Total histone H3 was used as loading control. H3K36me3 exposure = 20 s; H3 total exposure = 20 s. (**F**) The bars in the chart represent normalized H3K36me3 (versus total histone H3) values relative to Sh-*GFP* controls. Mean values ± SE from independent biological replicates (*n* = 4 for Sh4-*CHD8* and *n* = 6 for the other samples) are plotted. A *t*-test for two mean populations was performed. *P ≤0.05. (**G**) The heatmap represents gene set enrichment *P*-values in –log10 scale for all genes losing H3K36me3 (as in D) following *CHD8* knockdown. Gene lists related to ASD, neurodevelopment, co-expression modules in brain and intolerance to loss of function were tested for enrichment as described in the Materials and Methods. The full gene list description and enrichment results are available in Supplementary Table S1.

ChIP-seq reads were aligned on the human genome reference assembly GRCh38 using BWA (version 0.7.15) (43). Aligned reads were filtered to discard unmapped, multiply mapped, PCR duplicate reads (Picard tools MarkDuplicates version: 2.3.0, Picard Toolkit. 2019. Broad Institute, GitHub Repository http://broadinstitute.github.io/picard/) along with low quality alignments (samtools view -q 1, samtools version 1.71.7) (44). Peak calling was performed with MACS2 (version 2.1.0) using a minimum FDR (false discovery rate; Benjamini–Hochberg adjusted *P*-value) threshold of 0.00001. The same settings with the addition of the -broad option were used for H3K27me3 and H3K36me3 marks (45). Peaks localized to blacklisted regions (http://mitra.stanford.edu/kundaje/akundaje/release/blacklists/) or unplaced contigs were filtered out. Narrow peaks (for H3K27ac, H3K4me1, H3K4me2 and H3K4me3 histone marks) closer than 350 bp were merged into a single peak [BEDTools merge (version 2.25.0)]. Peaks were considered common between replicates if they overlapped by at least 50% of the length of the shortest peak [BEDTools intersect (version 2.25.0)], then extended coordinates were maintained and used in downstream analyses. CHD8 ChIP-seq reads from (11) were realigned [BWA (version 0.7.15)] to the reference GRCh38, and peaks were called with the same procedure as used for the narrow histone marks, with a default FDR threshold of 0.05 as only peaks identified by all three antibodies were retained. GENCODE v.26 was used for peak annotation (46). Genes losing H3K4me1, H3K27ac and H3K36me3 peaks in *CHD8* knockdown were tested for enrichment of Gene Ontology (GO) terms with the enricher function from clusterProfiler [version 3.10.1 (47)] with Benjamini–Hochberg adjusted *P*-value cut-off of 0.05. The full list of slimGO terms used was downloaded from Ensembl BioMart on September 9, 2019. Enrichment of the same gene list was assessed on custom gene sets by using one-tailed Fisher's exact test. Custom gene sets were derived from public databases and publications of interest (Supplementary Table S1). Only non-redundant gene lists enriched at *P*-value <0.01 are shown in Figure 1G; complete enrichment results are available in Supplementary Table S1. Combining the histone mark enrichment patterns over the genome, 10 chromatin states were identified for control (Sh-*GFP* and Sh-*GFP*2) using ChromHMM (version 1.14) (48). The states were manually annotated according to the literature. The number of peaks called in these regions in both replicates for each histone mark was counted using BEDTools intersect, version 2.25.0 (49), and the difference between control and *CHD8* knockdown was calculated. The total number of peaks for each histone mark as a percentage was plotted as a heatmap in control cells (Figure 1B, left); the difference (Figure 1B, right) refers to the percentage of control peaks-*CHD8* KD peaks. The number of peaks per mark called in each chromatin state was tested with a two-sided *t*-test (python 3.6 scipy.stats.ttest_ind) considering two replicates in both control and *CHD8* knockdown. Differential enrichment analysis for H3K36me3 was performed for three *CHD8* knockdown samples (Sh1-*CHD8*, Sh2-*CHD8* and Sh4-*CHD8*) and two controls (Sh-*GFP* and Sh-*GFP2*) with DiffBind [version 2.14, (50,51)] and DESeq2 for the differential analysis, using peaks previously called by MACS2, present in at least two samples. Peaks with FDR <0.05 were considered differentially enriched. The volcano plot in Figure 1 was plotted with ggplot2 [version 3.3.2 (52)]. To generate the metagene profiles with deepTools, version 3.2.1 (53), ChIP-seq samples were normalized to INPUT with the SES method (54). Enrichment was calculated in 10 bp bins over the gene body, scaled to 5 kbp and 2 kbp up- and downstream of the gene. In order to minimize noise in the metagene profile plots, a stringent set of filters was applied to protein-coding genes before plotting: a minimum length of 2 kbp, a minimum distance of 4 kbp from other genes and absence of other features on the opposite strand, leading to a set of 9442 protein-coding genes. For each histone mark, only genes with enrichment were plotted (at least one non-zero bin). These genes were divided into three groups based on the CHD8 binding enrichment pattern via k-means with deepTools plotProfile command. To complement the statistical hypothesis testing, paired Cohen's d effect size statistics were calculated between groups along the entire region (55). The 99% simultaneous confidence intervals were constructed controlling FWER (Bonferroni correction). CHD8-binding site profiles were calculated with deepTools computeMatrix reference point and plotted with deepTools plotProfile. Visualization of enrichment tracks for chromatin was performed with the Integrative Genomic Viewer [IGV, version 2.4.9 (56)].

### RNA-seq and AS data analysis

Raw reads obtained from Sugathan *et al.* (11) for corresponding samples (Sh-*GFP*, Sh-*GFP2*, Sh1-*CHD8*, Sh2-*CHD8* and Sh4-*CHD8*) were used to calculate transcript abundance by kallisto (version 0.44.00) (57) on GENCODE v.26 transcripts. Per-transcript RSEM-normalized read counts from RNA-seq of sh-*hnRNPL* and control samples in the HepG2 and K562 human cell lines were obtained from ENCODE (IDs: ENCSR155BMF and ENCSR563YIS). Transcripts per million (TPM) were used to plot expression levels. RNA-seq libraries from *hnRNPL* small interfering RNA (siRNA)-treated samples (si-*hnRNPL*-C) and si-scrambled control (si-SCR) were obtained from total RNA isolated using TRIZOL (Invitrogen) and treated with DNase (AMBION). RNA quality was assessed by Agilent bio-analyzer (RNA integrity number from 7.8 to 9.2). A stranded Illumina library was prepared according to the Illumina Stranded mRNA Prep manufacturer's protocol starting from 100 ng of purified total RNA. Samples was barcoded using the IDT for Illumina RNA UD Indexes, Ligation kit (Illumina). The library quality was checked by the DNA 1000 Kit and the 2100 Bioanalyzer instrument (Agilent Technologies). The 100 bp paired-end sequencing was performed by the Novaseq 6000 System (Illumina) at the Genomics Facility at the Italian Institute of Technology (IIT, Genova, Italy), obtaining ∼50–60 million reads per samples. SUPPA (version 2.3) was used to calculate the percentage spliced-in (PSI) value per splicing event with an empirical method (58). Significant splicing events were selected for a *P*-value <0.05 and ΔPSI >0.2. The same raw reads from Sugathan *et al.* (11) were aligned with STAR (version 2.6) (59) with default parameters on the GRCh38 reference, to analyze the AS with rMATS [version 3.1.0 (60)]. As the two AS analysis methods are complementary (https://github.com/comprna/SUPPA/issues/47), both were included in the analysis. To check the overlap of splicing events with chromatin marks, the spliced in/out exon coordinates were intersected with the coordinates of the histone mark peaks. AS events were represented in sashimi plots via ggsashimi (version 0.4.0) (61) using GENCODE annotation v.33 as a reference (46).

Raw reads from Suetterlin *et al.* (14) derived from P5 murine cortices (GSE81103; samples P5HET 1, P5HET 2, P5WT 1 and P5WT 2), along with reads from Sood *et al.* (62) from differentiated murine NPCs (GSE155217; samples RNA_WT_NPC_rep1, RNA_WT_NPC_rep2, RNA_CHD8_Het_NPC_rep1 and RNA_CHD8_Het_NPC_rep2) were processed as previously described and used for the validation analyses (Supplementary Figure S10). Murine genes derived from these two datasets were converted to the corresponding human orthologs by employing a conversion table (containing the Ensembl IDs for both species) obtained from BioMart (http://www.ensembl.org): GRCh38.p13 and GRCm39 datasets (Release 103) were used for the conversion (63).

### RBP motif pattern matching

Matches to known motifs for human RBPs, derived from the CISBP-RNA database (64), were obtained with the Biopython package v1.78 (65), using 95% similarity and 0.001 false-positive rate as thresholds. Sequences on which the match was performed were obtained by considering the 100 nt upstream and downstream of exons involved in a splicing event, extracted via the BioMart biomaRt R package (66).

### ChIP qPCR

Candidate genes for ChIP quantitative PCR (qPCR) experimental validation were chosen on genomic regions displaying both a significant H3K36me3 enrichment loss and at least one significant differential splicing event (resulting from SUPPA v2.3 RNA-seq analysis, 0.30 ΔPSI threshold). Genes displaying the highest signal variation in both ChIP-seq and RNA-seq were prioritized. FASTA sequences of genomic regions displaying significant H3K36me3 peak loss were retrieved and used as a template for ChIP qPCR primer design with the NCBI Primer-BLAST tool (https://www.ncbi.nlm.nih.gov/tools/primer-blast/), and an *in silico* specificity screen was performed with NCBI BLAST (https://blast.ncbi.nlm.nih.gov/Blast.cgi) and the UCSC In-Silico PCR tool (https://www.genome.ucsc.edu/cgi-bin/hgPcr). Amplicon size was tested by an electrophoretic gel run, and sequence specificity was verified by Sanger sequencing (Eurofins). qPCR analysis was performed using 200 pg of ChIP DNA and an equal amount of unenriched INPUT DNA, reaction volume 10 μl with iTaq™ Universal SYBR® Green Supermix (Biorad) using the recommended thermocycling parameters on Thermal Cycler C1000 CFX384 or CFX96 (Biorad). qPCR analysis was carried out with CFX Manager v3.1 Software. At least two technical replicates of qPCR were analyzed, with intra-assay variation <0.5 Cq. Two gene desert regions hGD12 (chr12:61273372–61273435) and hGD4 (chr4:187946873–187946954) were used as negative controls. Fold enrichment over INPUT was calculated using the 2^–ΔΔCq^ method. Depending on the condition, two or three biological replicates were analyzed for each cell clone. Error bars in the graphs represent the standard error of the mean (SEM).

### RNA extraction, reverse transcription and semi-quantitative PCR

Candidate genes for differential splicing experimental validation were prioritized among ChIP qPCR targets, as described above. For exon skipped events (SEs), FASTA sequences of invariant upstream and downstream exons (relative to the exon involved in the differential event) were retrieved from GENCODE annotation v.33, employing IGV (version 2.4.9), and used as a template for forward and reverse primer design, respectively, in order to amplify ‘spliced-in’ and ‘spliced-out’ transcript isoforms. Total RNA was extracted from iPSC-derived NPC lines using TRIZOL reagent (Invitrogen) following the manufacturer's instruction. DNA contamination was removed by treating the samples with DNase I (Invitrogen) and RNase inhibitor SUPERase (Invitrogen) for 30 min at 37°C followed by purification with the RNase Mini Kit (Qiagen). RNA quality was evaluated by an agarose gel electrophoretic run and Agilent 2100 Bioanalyzer. A 1 μg aliquot of RNA was retro transcribed using the SensiFAST cDNA Synthesis Kit (Bioline). The resulting cDNA was used to perform semi-quantitative PCR by employing the Phusion Green Hot Start II High-Fidelity PCR Master Mix (Thermo Scientific). TATA-binding brotein (TBP) was used as reference gene. PCR products were separated by an electrophoretic run on a 2% agarose gel. The ΔPSI of target transcripts was evaluated by densitometric analysis using ImageJ software (version 1.46r) and relativized to the Sh-*GFP* line as the reference sample. Data are presented as the mean ± SEM of calculated PSI value per splicing event. One-tailed *t*-test was performed using Excel; the significance level was reported as not significant (NS) *P* >0.05, *P ≤0.05, **P ≤0.01, ***P ≤0.001, ****P ≤0.0001.

### Acidic extraction and western blot analysis

The hiNPCs were washed with PBS and resuspended in 100 μl of extraction buffer [10 mM HEPES pH 8, 10 mM MgCl_2_, 0.1 mM EDTA pH 8, 0.1 mM DTT and halt protease and phosphatase inhibitor cocktail (Life Technologies)]. Samples were centrifuged at 5000 rpm for 10 min at 4°C to remove the cytosolic fraction. Nuclear pellets were resuspended in 0.2 N HCl and put in rotation at 4°C overnight. After centrifugation at 4000 rpm for 10 min at 4°C, supernatants containing nuclear proteins were recovered. Proteins were quantified by Bradford Protein Assay Kit (Sigma-Aldrich). Protein samples were separated by 4–12% Bis–Tris Protein Gels (Thermo Fisher) and transferred on an Amersham™ Protran™ 0.45 μm nitrocellulose (GE-Healthcare) membrane. Membranes were blocked with 5% w/v non-fat dried milk and incubated with the following primary antibodies: anti-CHD8 (NB100-60417, Novus Biologicals) (1:1000), anti-HSP90 (4874S, Cell Signaling Tech.) (1:5000), anti-histone H3 (1:1.000) (4499, Cell Signaling Tech.) and anti-histone H3K36me3 (1:1.000) (Ab9050, Abcam). Proteins were detected using horseradish peroxidase-conjugated secondary antibodies anti-rabbit IgG 1:7500 (GTX213110-01, GeneTex) and visualized by ECL Select WB detection reagent (GE Healthcare) following the manufacturer's instructions. Signal quantification was performed with Imagelab software (BIORAD, version 5.2.1).

### Statistical analysis

The analysis of target proteins differences in western blotting experiments was evaluated by performing an unpaired, one-tailed *t*-test. In all *t*-tests, the significance level was set to 0.05. Data were represented as mean ± standard deviation (SD). The significance level was reported as: NS *P* >0.05, *P ≤0.05, **P ≤0.01, ***P ≤0.001.

### Cellular fractionation

Cell fractionation was performed using the protocol described in (67) with minor modifications. Briefly, 50 × 10^6^ cells were resuspended in 5 ml of Hypotonic lysis buffer [HLB final component concentrations: 10 mM Tris (pH 7.5), 10 mM NaCl, 3 mM MgCl_2_, 0.3% (v/v) NP-40 and 10% (v/v) glycerol)] supplemented with PI and phosphatase inhibitor (PhI). The suspension was incubated on ice for 8 min prior to centrifugation at 4°C for 10 min at 800 *g*. The supernatant with the cytoplasmic fraction was kept separate from the pellet, containing the raw nuclear fraction (RNF). The RNF was then washed three times with HLB and centrifuged for 2 min at 800 *g* at 4°C. The pellet was resuspended in 2.5 ml of Nuclear lysis buffer [NLB, final component concentrations: 20 mM Tris (pH 7.5), 150 mM KCl, 3 mM MgCl_2_, 0.3% (v/v) NP-40 and 10% (v/v) glycerol] supplemented with PI and PhI. Samples were sonicated using Q700 (Qsonica) prior to centrifugation at 18000 *g* for 30 min at 4°C. The supernatant was collected and used as the nuclear fraction (NF) for immunoprecipitation and western blot analysis. The NF protein concentration was quantified by Pierce BCA Protein Assay Kit (Life Technology) using bovine serum albumin for the standard curve (Sigma). For western blot analysis, NF proteins were heated with SDS-loading buffer LDS sample buffer (Invitrogen) containing 5% Bolt sample reducing agent (Life Technologies) at 92°C for 10 min. Samples were loaded on 4–12% Bis–Tris protein gels (Thermo Fisher) with Protein Marker (Euroclone) and separated by electrophoresis. After electrophoresis, the proteins were transferred to nitrocellulose membranes (GE-Healthcare). Blotted membranes were incubated overnight with primary antibodies [CHD8 NB100-60417 (Novus Biotechnology), hnRNPL D-5 (sc-48391, Santacruz), SETD2 (38633, SAB), PARP1 (9542, Cell Signaling Tech.), Lamin A/C (sc-376248, Santacruz), GAPDH (ABS16, Merck), HSP90 3C9 (BSM-51215M, Bioss) histone H3 (trimethyl K36) (Ab9050, Abcam) and histone H3 (4499S, Cell Signaling Tech.)], then washed three times with PBS with 0.1% Tween and incubated for 1 h at 4°C with the specific secondary antibody.

### Immunoprecipitation and western blot analysis

For each immunoprecipitation, 1 mg of total protein in the nuclear fraction was incubated overnight rotating at 4°C with 1 μg of the primary antibody specific for the target protein. For CHD8 immunoprecipitation, NB100-60417 and NB100-60418 (Novus Biotechnology), for hnRNPL D-5 (sc-48391, Santacruz) and for SETD2 (38633, SAB) were used, while rabbit IgG isotype control (10500C, Life) or mouse IgG (10400C, Invitrogen) were used as controls. Protein A–Sepharose (CL-4B euroclone) or Protein G-Sepharose (GE-Healthcare) beads were pre-cleared by incubating them with the NF for 45 min rotating at 4°C. Beads were then removed and the cleared NF (CNF) was treated for 15 min at 37°C with 100 μg/ml RNase A (Invitrogen) or 2 U/ml DNase I (Invitrogen), or otherwise used directly for the overnight incubation with the primary antibody (NF with antibody, NFA). On the other hand, CNF was incubated overnight with primary antibody in the absence (not treated condition NT) or presence of 50 μg/ml ethidium bromide (EtBr; Sigma-Aldrich). All the different NFAs were finally incubated with beads for 45 min, rotating at 4°C. Bound complexes were then washed three times with NLB buffer. For western blot analysis, dried Sepharose bead complexes were eluted with SDS-loading buffer LDS Sample Buffer (Invitrogen) containing 5% Bolt sample reducing agent (Life Technologies) at 92°C for 10 min. Samples were loaded on 3–8% Tris-acetate protein gels (Thermo Fisher) with Protein Marker (Euroclone) and separated by electrophoresis. After electrophoresis, the proteins were transferred to nitrocellulose membranes (GE-Healthcare). Blotted membranes were incubated overnight with primary antibodies [CHD8 NB100-60417 (Novus Biotechnology), hnRNPL D-5 (sc-48391, Santacruz) and SETD2 (38633, SAB)], then washed three times with PBS with 0.1% Tween and incubated for 1 h at 4°C with the specific secondary antibody.

### Sample preparation and mass spectrometry

The co-immunoprecipitated samples were loaded on 10% SDS-polyacrylamide gel electrophoresis (PAGE) gel and run for ∼1 cm. Gels were then stained with Coomassie and the entire stained area was excised as one sample. Excised gel bands were cut into small pieces (∼1 mm^3^) and subjected to reduction and alkylation with 10 mM DTT and 55 mM iodoacetamide, respectively. Gel pieces were then washed in water, dehydrated with acetonitrile (ACN) and dried in a speed-vac. Gel plugs were re-hydrated with 50 mM NH_4_CO_3_ solution containing 12.5 ng/ml trypsin (Promega) on ice for 30 min. The digestion continued at 37°C overnight. The supernatant was collected, and the peptides were sequentially extracted from the gels with 30% ACN with 3% trifluoroacetic acid (TFA) and 100% ACN. All the supernatants were combined and dried in a SpeedVac. The tryptic peptides were then acidified with 1% TFA to a pH <2.5, desalted on C18 stage tips and resuspended in 20 μl of 0.1% formic acid buffer for liquid chromatography–tandem MS (LC-MS/MS analysis). Peptides were separated on an Easy-nLC 1200 high-performance liquid chromatography (HPLC; Thermo Fisher) system by 85 min gradients with a 400 nl/min flow rate on a 25 cm column with an inner diameter of 75 μm packed in-house with ReproSil-Pur C18-AQ material (3 μm particle size, Dr Maisch, GmbH). The gradient was set as follows: from 5% to 25% over 52 min, from 25% to 40% over 8 min and from 40% to 98% over 10 min at a flow rate of 400 nl/min. Buffers were 0.1% formic acid in water (A) and 0.1% formic acid in ACN (B). Peptides were analyzed in an Orbitrap Fusion Tribrid mass spectrometer (Thermo Fisher) in data-dependent mode, with a full-scan in the Orbitrap performed at 120 000 FWHM resolving power (at 200 *m/z*), followed by a set of (higher energy collision dissociation) MS/MS scans over a 3 s cycle time. The full scans were performed with a mass range of 350–1100 *m/z*, a target value of 1 × 10^6^ ions and a maximum injection time of 50 ms. The MS/MS scans were performed at a collision energy of 30%, 150 ms of maximum injection time (ion trap) and a target of 5 × 10^3^ ions.

### Mass spectrometry data analysis and processing

Raw files were searched using Proteome Discoverer software v.2.2.0 (Thermo Fisher Scientific). Peptide searches were performed against the *in silico* digested UniProt Human database (downloaded July 2019) and a database containing common contaminants. Trypsin/P was chosen as the enzyme with five missed cleavages. Static modification of carbamidomethyl (C) with variable modification of oxidation (M) and acetylation (protein N-term) were incorporated in the search. The MASCOT search engine [v.2.6.2 (MatrixScience)] was used to identify proteins, using a precursor mass tolerance of 10 ppm and a product mass tolerance of 0.6 Da. The FDR was set to 1% at both the peptide and protein level. The results were filtered to exclude potential contaminants. Peak intensities of peptides were normalized on the average of the specific protein abundance within each sample (68). Log2-normalized intensities of single peptides were averaged through replicates and by subtracting the averaged values of IgG to IP samples; the fold change (FC) was calculated for each peptide. Then, the FC of each protein was calculated by averaging the FC of all peptides assigned to each protein. CHD8-binding partners were detected by comparing fold enrichment of the IP samples versus IgG control (IP/IgG). Statistical significance was assessed using Student's *t*-test (two-tailed, two-sample unequal variance, Excel). In the case of a single replicate, the enrichment of each peptide was calculated by subtracting the log2-normalized intensities of the IgG to the IP sample. Statistical significance was calculated as previously described (Student's *t*-test).

### Targeted siRNA knockdown for hnRNPL

A total of 3 ×10^6^ hiNPCs were electroporated with 200 pM siRNA oligos using the Lonza Nucleofector® 2b device and a custom-made electroporation buffer (5 mM KCl, 15 mM MgCl_2_, 10 mM glucose and 120 mM K_2_HPO_4_/KH_2_PO_4_ pH 7.2). Knockdown efficiency was determined after 48–72 h from electroporation. The siRNA oligos targeting the hnRNPL gene (three unique 27-mer and one universal scrambled negative control) were purchased from OriGene (SR302174). Proteins for western blotting were extracted using RIPA buffer (ThermoScientific), and sonicated using Q700 (Qsonica) prior to centrifugation, while total RNA was obtained by using TRIZOL (Invitrogen).

## RESULTS

### *CHD8* suppression significantly affects transcriptional elongation chromatin states

To assess the functional consequences of *CHD8* suppression on chromatin organization, we resorted to a previously characterized control iPSC-derived NPC line, GM8330-8, and its derivatives where ∼50% reduction in *CHD8* was obtained by lentiviral-mediated delivery of shRNAs (11). In these model systems, we analyzed six different histone modifications using ChIP-seq, specifically interrogating transcriptionally active (H3K4me2, H3K4me3, H3K27ac and H3K36me3) and repressed regions (H3K27me3) as well as active/poised enhancers (H3K4me1 and H3K27ac). For each of the six histone marks, three independent shRNAs targeting the coding sequence of *CHD8* (Sh1, Sh2 and Sh4) and two technical replicate controls against the *GFP* sequence were used (Figure 1A). Sh1-*CHD8*, Sh2-*CHD8* and Sh4-*CHD8* presented nearly comparable levels of *CHD8* at ∼50% of their physiological levels, thus precisely mimicking the human haploinsufficiency condition (see Supplementary Figure S1A and B for transcript and protein level) (11). Importantly, for *CHD8*-knockdown models as well as for *GFP* controls, genome-wide transcriptomic data and CHD8-binding sites were available (Figure 1A) (11).

ChIP-seq experiments were conducted to obtain on average 40 million reads for narrow marks (H3K4me3/me2/me1 and H3K27ac) and 60 million for broad histone marks (H3K36me3/K27me3) and INPUT samples. After mapping and filtering, an average of 42 308 peaks per sample were identified (Supplementary Figure S1C, D), and showed the expected enrichment pattern and metagene profiles at the transcriptional start site (TSS: H3K4me3/me2/me1 and H3K27ac), the gene body (H3K36me3) of actively transcribed genes or on larger genomic regions spanning transcriptionally silent gene units (H3K27me3), and the expected enrichment correlation with ENCODE public datasets (Supplementary Figure S1E, F) (69,70).

Upon *CHD8* suppression, H3K4me1, H3K27ac and H3K36me3 presented a substantial decrease in the number of peaks (37.44, 38.45 and 47.82%, respectively) compared with the control (intersection of Sh-*GFP* and Sh-*GFP2*) (Supplementary Figure S2A). However, some level of replicate heterogeneity was observed, especially for H3K4me1 and H3K27ac, while H3K36me3 presented a more consistent and considerable reduction (Supplementary Figures S1D–F and S2A). Importantly, the third biological replicate Sh1-*CHD8*, presenting less efficient *CHD8* transcription suppression (Supplementary Figure S1A) (11), but stronger CHD8 protein reduction (Supplementary Figure S1B), confirmed impaired H3K36me3 enrichment (Supplementary Figure S2A). In addition, Sh2-*CHD8* and Sh-*GFP* independent ChIP-seq datasets (generated in a different laboratory from the previous set) again sustained the conclusion that *CHD8* suppression was associated with a decreased H3K36me3 enrichment (Supplementary Figure S2B).

By combined analysis of the histone mark enrichment through ChromHMM (48), we defined 10 types of genomic regions with a specific chromatin state in control hiNPC [1, transcriptional initiation (H3K4me3, H3K4me2, H3K4me1 and H3K36me3); 2, transcriptional elongation (high H3K36me3); 3, transcribed weakly (low H3K36me3); 4, strong enhancer (H3K4me2, H3K4me1 and H3K27ac); 5, weak/poised enhancer a (H3K4me2 and H3K4me1); 6, weak/poised enhancer b (H3K4me1 and H3K27ac); 7, active promoter (H3K4me3, H3K4me2, H3K4me1 and H3K27ac); 8, inactive/poised promoter (H3K27me3, H3K4me3, H3K4me2 and H3K4me1); 9, Polycomb repressed (H3K27me3); and 10, heterochromatin/low signal (no enrichment)] (Figure 1B, left). To identify states affected by *CHD8* knockdown, we then compared the histone marks (peak counts for each histone mark within each chromatin state) across two conditions, i.e. controls versus *CHD8* knockdown (Sh2-*CHD8* and Sh4-*CHD8*) (Figure 1B, right) and detected transcriptional elongation, strong–weak/poised enhancer and active promoter as the chromatin states most affected by *CHD8* suppression.

To further dissect these results, we compared the number of peaks for each histone mark in controls and *CHD8* knockdowns within each chromatin state. While differences between biological replicates did not support a statistically significant variation at enhancer and promoter states (Supplementary Figure S3A, B), the number of peaks decorated by histone H3K36me3 at genomic regions involved in transcriptional elongation was significantly lower in Sh2–Sh4 *CHD8* (*P*-value <0.05, *t*-test) compared with controls (Figure 1C). Within the genomic regions involved in transcriptional elongation, H3K36me3 reduction affected ∼50% of all expressed protein-coding genes in human NPCs (out of the protein-coding genes with >2 TPM, 5447 lost H3K36me3 while 6451 were not affected). Additionally, genes that lost H3K36me3 following *CHD8* haploinsufficiency were significantly longer (90290 versus 60902 bp) and composed of more exons (7.36 versus 6.20) (not shown) compared with the unaffected genes. Independent quantitative analysis using DiffBind and western blot further supported a critical reduction of H3K36me3-bound regions in the three Sh1–Sh2–Sh4 *CHD8* knockdown clones compared with control Sh-*GFP* (Figure 1D-F). On the contrary, metagene profiles, effect size and DiffBind analyses reported no substantial difference for H3K27ac and H3K4me1 marks (Supplementary Figure S3C–H). A composite heatmap of all genes presenting reduced H3K36me3 following *CHD8* suppression versus unaltered genomic location is shown in Supplementary Figure S4. Genes with reduced H3K36me3 following *CHD8* suppression were strongly enriched for ‘constrained’ genes [intolerant to loss-of-function mutations, gnomAD (71)], ‘FMRP targets in brain’ (72), SFARI ASD genes (https://gene.sfari.org/about-gene-scoring/), ‘essential genes’ [required for a cell's survival (73)] and the ‘M3 co-expression module’ (6), whose expression peaks early during nervous system development (Figure 1G; Supplementary Table S1).

### *CHD8* suppression-dependent reduction in histone H3 Lys36 trimethylation impacts on CHD8-bound genes

By overlaying human CHD8-binding sites on the previously established chromatin states (Figure 1B), we confirmed that CHD8 was confined to active promoters (90.11%) and, less prominently, to enhancers [strong, weak/poised a and b (3.18, 3.00 and 1.34%)] (Supplementary Figure S5A) (11). As previously reported (74), CHD8 binding correlated with higher histone H3K36me3 (Supplementary Figure S5B, E) and H3K4me3 (Supplementary Figure S5C, F) enrichment of metagene profiles as well as elevated RNA expression levels compared with CHD8-unbound genes (Supplementary Figure S5D).

**Figure 2.**
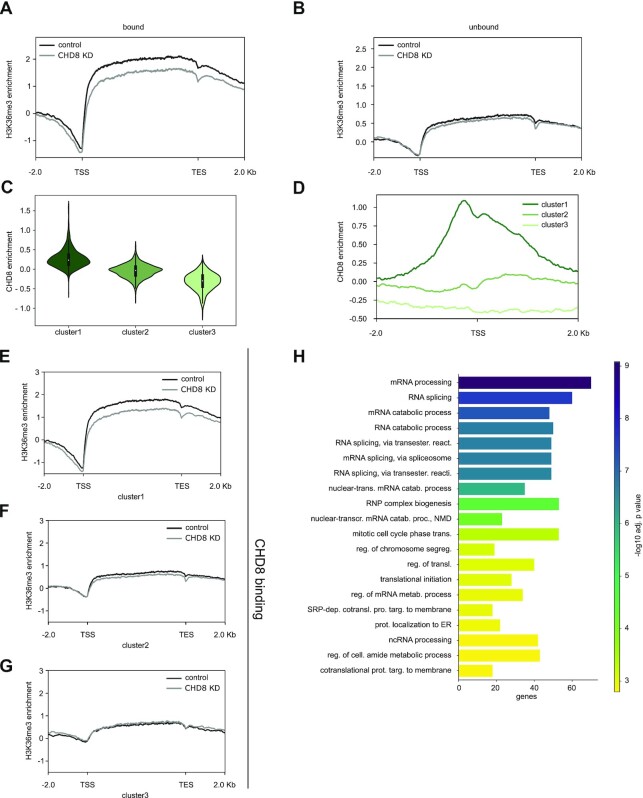
*CHD8* suppression correlates with reduced H3K36me3 enrichment preferentially at CHD8-bound genes. (**A**, **B**) Metagene profiles display the average of histone H3K36me3 enrichment (scaled log2 ratio of normalized ChIP value over INPUT control; see also the Materials and Methods) in a region of ± 2 kbp upstream of the TSS and downstream of the TES, calculated for control (black line) and *CHD8* knockdown (gray line) hiNPCs and for CHD8-bound (#988) (A) and CHD8-unbound genes (#4205) (B). The difference between H3K36me3 enrichment in control and CHD8 knockdown is significant for CHD8-bound genes, but not for CHD8-unbound genes (paired Cohen's d effect size statistics). (**C**) The violin plots represent the level of CHD8 binding enrichment [log2(ChIP/INPUT)] for three groups of genes as clustered by k-means. Cluster #1 composed of 1239 genes shows high CHD8 enrichment [mean log2(ChIP/INPUT) = 0.27], cluster #2 composed of 2429 genes shows medium to low CHD8 enrichment [mean log2(ChIP/INPUT) = –0.04] and cluster #3 composed of 1380 genes displays negligible CHD8 enrichment [mean log2(ChIP/INPUT) = –0.31]. (**D**) Metagene profiles show the average of CHD8 binding enrichment in a region of ± 2 kbp around the TSS, calculated for the three clusters #1, #2 and #3 as described in (C). (**E–G**) Metagene profiles display the average of histone H3K36me3 enrichment [log2(ChIP/INPUT)] in a region of ± 2 kbp upstream of the TSS and downstream of the TES, calculated for control (black line) and CHD8 knockdown (gray line) hiNPCs for each of the three clusters identified in (C). The difference between H3K36me3 enrichment in control and *CHD8* knockdown is significant for cluster #1, but not for clusters #2 or #3 (paired Cohen's d effect size statistics, see Supplementary Figure S6B–D). (**H**) Bar plot presenting the top 20 biological process GO terms significantly enriched in genes belonging to clusters #1 and #2, with high/medium CHD8 binding enrichment in control hiNPCs and a lower H3K36me3 enrichment in *CHD8* knockdown (in E and F). Bars are colored according to –log10 (adjusted *P*-values) and the *x*-axis represents the number of genes per term.

Upon *CHD8* suppression, stringently defined (see the Materials and Methods) CHD8-bound genes appeared to be more sensitive than CHD8-unbound genes (988 and 4205, respectively), presenting a significantly reduced H3K36me3 enrichment profile as confirmed by the effect size analysis (Figure 2A, B; Supplementary Figure S6A). Notably, the reduction in histone H3K36me3 elicited by *CHD8* suppression was specific since histone H3K4me3, another histone modification enriched at TSSs of highly expressed genes (Supplementary Figure S7A, B), remained unaltered (Supplementary Figure S7C–E).

Clustering analysis based on CHD8-binding site enrichment in control hiNPCs identified three different clusters: cluster #1 composed of 1239 genes with high CHD8 enrichment [mean log2(ChIP/INPUT) = 0.27], cluster #2 composed of 2429 genes with medium to low CHD8 enrichment [mean log2(ChIP/INPUT) = –0.04] and cluster #3 composed of 1380 genes with negligible CHD8 enrichment [mean log2(ChIP/INPUT) = –0.31] (Figure 2C D). Strikingly, cluster #1 was strongly affected by CHD8 decline, displaying significantly reduced H3K36me3 levels across the gene body (Figure 2E; Supplementary Figure S6B). Clusters #2 and #3, instead, with poor CHD8 enrichment in control hiNPCs, displayed a correspondingly lower H3K36me3 enrichment, with no significant difference following *CHD8* suppression (Figure 2F, G; Supplementary Figure S6C, D). In conclusion, this analysis confirmed that CHD8-bound genes were strongly sensitive to CHD8 reduction, presenting a substantial and specific drop in H3K36me3 histone modification (Figure 2C–D; Supplementary Figure S6B). Functional enrichment of genes from clusters #1 and #2, i.e. CHD8-bound and losing H3K36me3 enrichment upon CHD8 suppression, highlighted GO biological process terms related to ‘mRNA processing’ and ‘RNA splicing’ (Figure 2H; Supplementary Table S1). Functional enrichment of genes from cluster #3 is also presented (Supplementary Figure S8).

### Reduction in histone H3 Lys36 trimethylation elicited by *CHD8* suppression alters RNA AS

To gauge the functional significance of H3K36me3 reduction observed following *CHD8* suppression, we then leveraged RNA-seq data from controls and *CHD8*-knockdown clones. As histone H3K36me3 seems to be correlated with high levels of RNA expression (Supplementary Figure S7A) (75), we reasoned that a decline in H3K36me3 levels would be associated with reduced RNA expression levels. Unexpectedly reduced levels of CHD8, and impaired H3K36me3 enrichment, did not correspond to a global difference in transcription in either CHD8-bound or CHD8-unbound genes (Supplementary Figure S9A–C).

**Figure 3.**
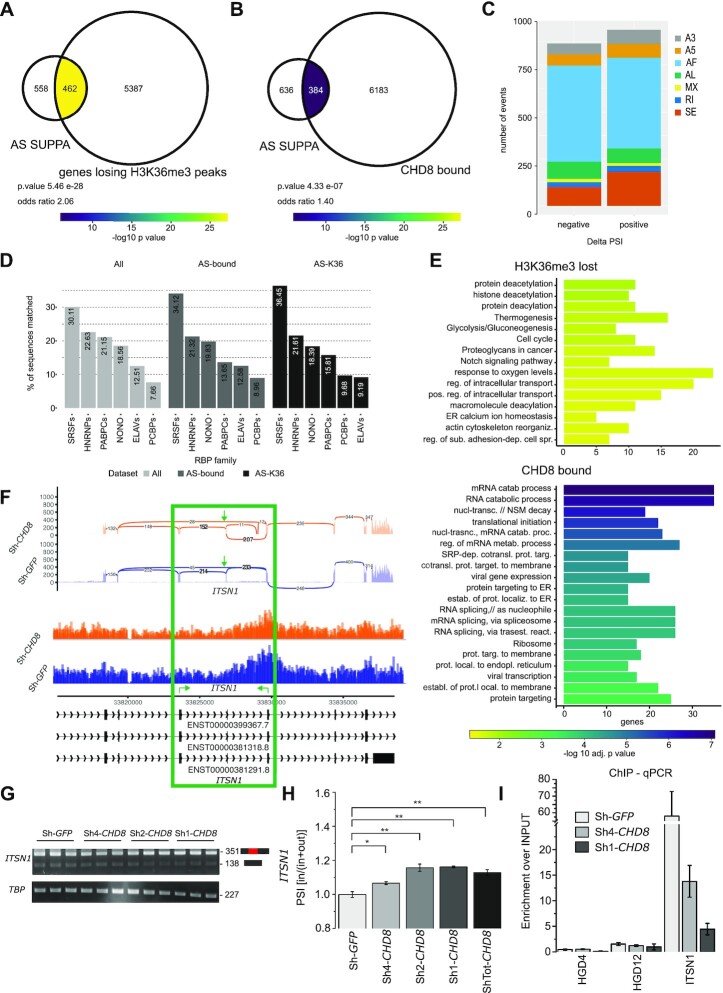
*CHD8* suppression-elicited reduction in H3K36me3 correlates with significant alterations in RNA AS. (**A**, **B**) Venn diagrams represent the overlap between genes losing H3K36me3 peaks following *CHD8* knockdown (losing H3K36me3) and genes presenting altered AS events as detected by SUPPA (AS SUPPA) (A), and the overlap between genes bound by CHD8 (CHD8-bound) and genes presenting altered AS events as detected by SUPPA (AS SUPPA) (B). The number of genes for each condition is indicated. The enrichment significance for each intersection is computed by Fisher's exact test and represented by colors. Color-coded key: –log10(*P*-value). The *P*-value and odds ratio are reported. (**C**) The stacked bar plot represents the 1862 differential AS events detected by SUPPA, distributed by event type. SE, skipped event; RI, retained intron; MX, mixed event; A3, alternative 3′; A5, alternative 5′; AF, alternative first exon; AL, alternative last exon. (**D**) The bar plot reports the overlap between sequences located ± 100 bp from all differentially spliced exons (all, light gray), differentially spliced and bound by CHD8 (AS-bound, gray) or differentially spliced and losing H3K36me3 (AS-K36, dark gray) and known RBP family motif matching. The six most representative RBP families are reported. The exact percentage of sequences matched is indicated within each bar. (**E**) The bar plot represents GO biological process and KEGG pathways terms significantly enriched in genes presenting altered AS events as detected by SUPPA and losing H3K36me3 peaks following *CHD8* knockdown (H3K36me3 lost, top panel) or bound by CHD8 (CHD8 bound, bottom panel). The bars are ordered according to adjusted *P*-value in –log10 scale; the *x*-axis represents the number of genes enriched for each term. (**F**) The image displays sashimi plots (top), chromatin tracks of H3K36me3 enrichment (middle) and ENSEMBL transcript IDs (bottom) for the *ITSN1* locus. Sh-*CHD8* samples versus Sh-*GFP* controls are color-coded (in orange and blue, respectively). Numbers of junction reads are depicted on the top panel. The green box and green arrows highlight the skipped exon event. Green arrows on the bottom panel show the location of primers used to analyze the skipped exon event. (**G**) Gel images represent PCR products obtained using *ITSN1* (top) and *TBP* reference primers (bottom). Using the *ITSN1* primer set, two amplicons are obtained corresponding to target exon inclusion (351 bp) and exon skipped (138 bp). (**H**) The bar graph reports Image-J PCR band quantification of *ITSN1* transcripts normalized using *TBP* as the reference gene. PSI (% spliced in) is obtained by calculating the ratio between *ITSN1* spliced in transcripts and the sum of spliced in plus spliced out events: PSI [in/(in + out)]. Means ± SE are shown. One-tail *t*-test was performed with NS *P* >0.05, *P ≤0.05, **P ≤0.01. (**I**) The bar plot shows CHD8 enrichment over the INPUT following CHD8-ChIP qPCR quantification. *ITSN1* genomic primers (green arrows in F) and gene desert control regions (*HGD4* and *HGD12*) were amplified for Sh-*CHD8* and Sh-*GFP* conditions.

Importantly, a significant proportion of genes losing H3K36me3 upon *CHD8* suppression [462 genes of which 176 (38.1%) had CHD8-binding sites in promoter or enhancer regions] presented altered AS profiles, as evidenced by two analysis approaches (Figure 3A, B; Supplementary Figure S9D–F) (74,76). We noted an almost equal distribution of the H3K36me3 peaks lost following *CHD8* suppression between exonic and intronic regions (data not shown).

The vast majority of altered AS events were categorized as ‘alternative first exon’ [994 (51.19%)] or ‘exon skipping’ [283 (15.71%)] (Figure 3C; Supplementary Figure S9F). For the ∼1000 genes for which differential splicing events were detected by SUPPA (Figure 3A, B), the proportion of events presenting positive or negative ΔPSI (ΔPSI = PSI_ctrl – PSI_KD) [937 events (52.03%) have positive values; 864 events (47.97%) have negative values] remained pretty similar. Interestingly, RBP motif matching of the ± 100 bp sequences adjacent to all differentially spliced exons (all), differentially spliced and bound by CHD8 (AS-bound) or differentially spliced and losing H3K36me3 (AS-K36) revealed enrichment for SRSFs, hnRNPs and ELAVs RNA-binding proteins, suggesting a possible regulatory role for these factors in the observed phenotype (Figure 3D). Among genes characterized by a reduction in H3K36me3 with concomitant splicing alterations, over-representation of GO terms and pathways related to ‘protein deacetylation’, ‘histone deacetylation’ and ‘protein deacylation’ (Figure 3E, top; Supplementary Table S1) was observed, while those bound by CHD8 and presenting altered splicing patterns following *CHD8* suppression were enriched for ‘mRNA catabolism’, ‘translational initiation’ and ‘regulation of RNA splicing’ (Figure 3E, bottom; Supplementary Table S1). The direct comparison of specific H3K36me3 lost peaks and exons presenting a differential splicing event revealed modest intersection (Supplementary Figure S9G). Nevertheless, at eight genomic coordinates, concomitant reduction in H3K36me3 and AS variation with ΔPSI >0.30 could be observed. Specifically, experimental validation at the *ITSN1* locus, the Intersectin 1 gene involved in endocytic membrane traffic and synaptic transmission (77,78), complex learning and memory formation (79) as well as previously implicated in schizophrenia (80), confirmed a clear reduction in H3K36me3 enrichment and a corresponding increase in the PSI ratio (spliced in/spliced in + spliced out) of the splicing variants in hNPCs presenting knockdown levels of *CHD8* (Figure 3F–I).

The aberrant AS phenotype was also mirrored in previously published murine datasets (14,62). Specifically, *Chd8* genetic ablation in P5 cortical regions (Supplementary Figure S10A, C, D, F) and mouse NPCs (mNPCs; Supplementary Figure 10B, C, E, F) elicited AS pattern alteration that closely resembled (numerically and in the AS subtype distribution) the one described in iPS-derived NPCs. For P5 cortical samples, 950 (59.90%) ‘alternative first exon’ and 191 (12.04%) ‘exon skipping’ events were detected. Similar values. i.e. 840 (52.01%) ‘alternative first exon’ and 240 (14.86%) ‘exon skipping’, were observed in mNPCs. The distribution of events with positive or negative ΔPSI also remained similar (51.01% positive and 48.99% negative ΔPSI in the P5 cortical samples, and 49.10% positive and 50.90% negative ΔPSI in mNPC samples) (Supplementary Figure S10A, B), thus supporting the hypothesis that *CHD8*/*Chd8* suppression generally correlates with AS defects, regardless of the species considered. Interestingly, although the genes presenting splicing alterations within each neuronal model system were generally different (GO terms/pathways presented in Supplementary Figure S10G–I), nevertheless significant overlaps for some of the intersections tested were identified (Fisher's test, Supplementary Figure S10C–F), suggestive of a core group of genes, half of which were bound by CHD8 (i.e. *ITSN1*, *CNOT2* and *MDM2*), involved in ‘neuronal differentiation’, ‘cell cycle’ and ‘DNA repair’, were strongly sensitive to splicing alterations.

### hnRNPL as a novel CHD8 interactor: bridging altered splicing to H3K36me3 enrichment

**Figure 4.**
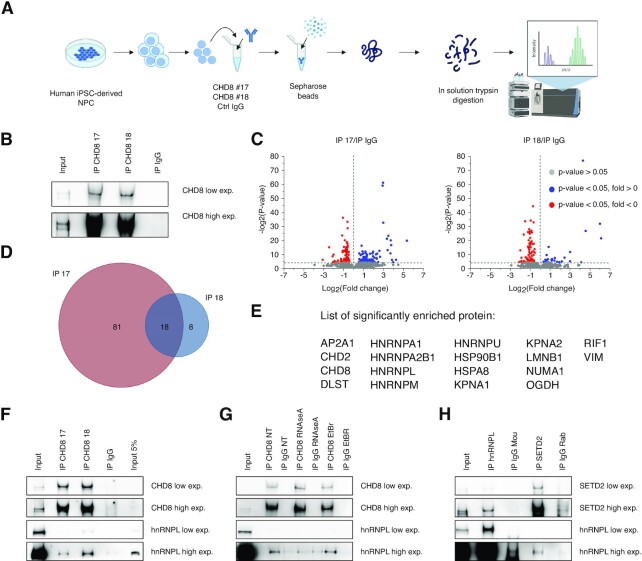
hnRNPL as a novel CHD8 interactor: bridging altered splicing to H3K36me3 enrichment. (**A**) Schematic representation of the MS/MS experimental design and approach used in this work (figure created in BioRender.com). Nuclei from hiNPCs (11) were separated from the cytoplasmic fraction. The protein of interest was isolated from the nuclear lysate by specific primary antibodies followed by incubation with Sepharose beads. CHD8 immunoprecipitated proteins were then processed by in solution trypsin digestion prior to MS/MS analysis. (**B**) Representative western blot images depict immunoprecipitation by endogenous, full-length CHD8 in nuclear extracts by two different antibodies CHD8 NB100-60417 (CHD8 #17) and NB100-60418 (CHD8 #18). A strong, reproducible enrichment compared with Input (Input, 15 μg of nuclear lysate) and rabbit IgG control (IgG) is evident. High exp, 30 s; low exp = 4 s. (**C**) The volcano plots show CHD8-interacting proteins, significantly differentially enriched compared with IgG controls. Significantly enriched proteins are in blue, significantly depleted proteins in red and non-significant proteins in gray. The threshold for significance is set at a *P*-value of 0.05. Three independent experiments were averaged and analyzed together for each condition. CHD8, the more represented and enriched peptide with each of the two antibodies, was removed from the plots to optimize visualization of interactors. (**D**) Venn diagrams represent the overlap between CHD8-interacting proteins identified by CHD8 #17 and CHD8 #18 antibodies. The analysis combines the statistically significant results from three independent biological replicates and two antibodies (Ab #17 A, B, C; Ab #18 A, B, C; see also Supplementary Figure S11). The number of proteins for each condition is indicated. The complete list of proteins is given in Supplementary Table S1. (**E**) Complete list of the 18 CHD8-interacting proteins identified by CHD8 #17 and CHD8 #18 antibodies. (**F**) Representative western blot images from co-immunoprecipitation experiments demonstrate interaction between endogenous CHD8 and hnRNPL. Immunoprecipitations were conducted with the two antibodies (IP CHD8 #17 and IP CHD8 #18). A strong, reproducible CHD8 enrichment compared with Input (Input, 15 μg of nuclear lysate, Input 5%, 0.75 μg of nuclear lysate) and rabbit IgG control (IgG) is evident. Co-immunoprecipitation of hnRNPL is clearly visible at high exposure. CHD8 high exp, high exposure = 60 s; CHD8 low exp, low exposure = 20 s. HnRNPL high exp, high exposure = 240 s; CHD8 low exp, low exposure = 75 s. (**G**) Representative western blot images showing immunoprecipitation of endogenous CHD8 in the nuclear extract with different treatments: RNase A (RNaseA), EtBr or no treatment (NT). CHD8 high exp, high exposure = 30 s; CHD8 low exp, low exposure = 10 s. HnRNPL high exp, high exposure = 60 s; hnRNPL low exp, low exposure = 4 s. (**H**) Representative western blot images report co-immunoprecipitation between hnRNPL (anti-mouse) and SETD2 (anti-rabbit) antibodies. Endogenous hnRNPL interacts with SETD2, as demonstrated by enrichment over mouse IgG (IP IgG Mou) and INPUT (15 μg of nuclear lysate). Reciprocal co-immunoprecipitation of endogenous SETD2 confirms the interaction, visible at high exposure, compared with IgG controls (IP IgG Rab). SETD2 high exp, high exposure = 20 s; SETD2 low exp, low exposure = 4 s. HNRNPL high exp, high exposure = 40 s; hNRNPL low exp, low exposure = 2 s.

**Figure 5.**
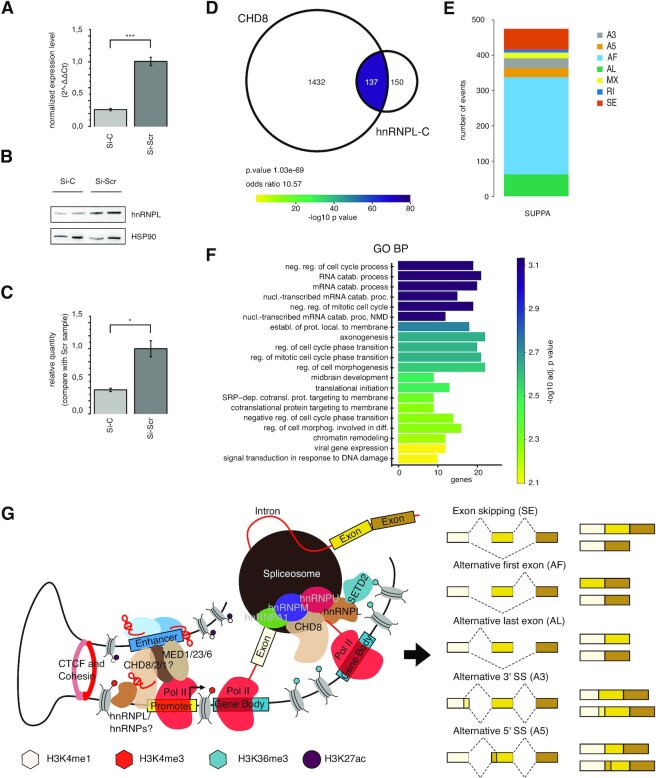
siRNA-mediated *hnRNPL* reduction causes AS changes that partly mirror those elicited by *CHD8* suppression. (**A**) The bar graph reports the normalized expression levels (2^−ΔΔCT^) of *hnRNPL* transcript following Si-*C* administration for 72 h in hiNPCs. Four different biological replicates per experimental condition are reported. Means ± SE are shown. One-tail *t*-test was performed, with ***P ≤0.001. (**B**) Representative western blot images illustrate total hnRNPL levels, comparing hiNPC exposed to siRNA against *hnRNPL* (Si-*C*) and control (Si-*Scr*). Two representative biological replicates per condition are presented. Comparable amounts of total protein were loaded as detected by the HSP90 loading control. (**C**) The bars in the chart represent normalized hnRNPL protein levels comparing hiNPCs exposed to siRNA against *hnRNPL* (Si-*C*) and control (Si-*Scr*). HnRNPL bands were normalized on HSP90 loading control. Mean values ± SE from four independent biological replicates are plotted. *T*-test for two mean populations was performed, *P ≤0.05. (**D**) Venn diagrams represent the overlap between genes presenting aberrant splicing following *CHD8* suppression and genes presenting altered AS events after siRNA-mediated reduction of hnRNPL (72 h post-electroporation). The AS events are detected by SUPPA. The number of genes for each condition is indicated. The enrichment significance for the intersection is computed by Fisher's exact test and represented by colors. Color-coded key: –log10(*P*-value). The *P*-value and odds ratio are reported. (**E**) The stacked bar plot represents the 473 differential AS events detected by SUPPA in hiNPCs subjected to siRNA against *hnRNPL* distributed by event type. Event types: SE, skipped event; RI, retained intron; MX, mixed event; A3, alternative 3′; A5, alternative 5′; AF, alternative first exon; AL, alternative last exon. (**F**) The bar plot represents GO biological process and KEGG pathway terms significantly enriched in genes presenting altered AS events as detected by SUPPA following *CHD8* and *hnRNPL* suppression (intersection in D). The bars are ordered according to adjusted *P*-values in –log10 scale; the *x*-axis represents the number of genes enriched for each term. (**G**) CHD8 functions at transcription initiation, elongation and regulation of AS. Schematic representation of the molecular mechanisms proposed to explain CHD8’s roles at promoters/enhancers and in the modulation of AS (figure created in BioRender.com). CHD8 interacts with hnRNPL, possibly through stabilizing RNA bridges (red hairpins). Thus, CHD8/hnRNPL association, possibly also recruiting the Mediator complex (106), might stabilize enhancer/promoter looping and transcriptional initiation. However, hnRNPL also solidly interacts with SETD2 at elongating RNAPII, thus implicating CHD8 in the regulation of RNA processing and AS. By comparing CHD8 and SETD2 MS experiments [our data and (39)], a number of other hnRNP interactors emerge, providing a functional link between SETD2, hnRNPs and CHD8 with elongating RNAPII, with functional consequences for the regulation of the splicing machinery. Differential alternative splicing events can be modulated by the CHD8/hnRNPL/SETD2 complex.

To shed light on the observed AS phenotype and to unveil how CHD8 controls H3K36me3 enrichment at the genes’ body, we characterized the CHD8 interactome by MS/MS analysis using two independent, immunoprecipitation-validated, antibodies (#17-N- and #18-C-terminal, see the Materials and Methods) on the hNPC nuclear-enriched fraction (Figure 4A, B; Supplementary Figure S11A). MS using the two different antibodies, and averaging the peptides obtained by three independent biological replicates, resulted in a list of new and previously reported [CHD7, histone H1 and POLR2 (81–84)] CHD8 interactors (Figure 4C, D; Supplementary Figure S11B, C; Supplementary Table S1). CHD8 #17 antibody yielded a higher number of immunoprecipitated and sequenced peptides (*n* = 99), compared with CHD8 #18 (*n* = 26). Combining the statistically significant results from three independent biological replicates and two antibodies (Ab #17 A, B, C; Ab #18 A, B, C), a list of 18 stringently defined protein interactors (Figure 4D, E) was obtained. These proteins were strongly enriched for terms related to ‘RNA binding’, ‘mRNA processing’ and ‘mRNA splicing, via spliceosome’ (Supplementary Figure S11D). Specifically, 5 out of the 18 stringently defined, new CHD8-interacting candidates belonged to the hnRNP RBP family already foreseen by motif analysis of the ± 100 bp sequences adjacent to all differentially spliced exons (Figure 3D), actively regulating AS, mRNA stabilization and transcriptional and translational processes (85) (Figure 4C–E). Importantly, independent Chd8 MS analysis on mouse embryonic stem cells (mESCs) identified a similar pattern of interacting proteins, with GO terms and KEGG (Kyoto Encyclopedia of Genes and Genomes) pathways associated with ‘spliceosome’ and ‘mRNA processing’ redundantly enriched between the two experimental models (Supplementary Figure S11E, F). Among CHD8-interacting proteins, hnRNPL, sequenced in both hNPCs and mESCs, was validated as a direct CHD8 interactor, not sensitive to DNase and EtBr treatments, in independent co-immunoprecipitation experiments (Figure 4F, G; Supplementary Figure S11G). However, RNase A treatment partially impacted the binding between CHD8 and hnRNPL (Figure 4G; Supplementary Figure S11H), thus suggesting a possible role for RNA molecules in the binding or functional stabilization of this interaction. Although not directly implicated in H3K36 methyltransferase activity, hnRNPL was previously described as SETD2 interactor and regulator (40). In fact, the previously reported binding between SETD2 and hnRNPL was also confirmed in our hNPC model (Figure 4H), suggesting a novel, indirect role for CHD8 in modulating SETD2-dependent H3K36me3 via hnRNPL, bridging AS changes with chromatin regulation. HnRNPL was shown to play a direct role in splicing regulation (86), binding to CA-rich RNA elements (87) and repressing cryptic exon inclusion (88). Thus, we asked whether hnRNPL suppression could correlate with dysfunctional splicing events mirroring CHD8 suppression. To gain a comprehensive view of hnRNPL-dependent AS events in hiNPCs, we electroporated either control or *hnRNPL*-targeting siRNAs [single oligos (A, B or C)] to our cells. Initial experiments to test siRNA efficiency showed a high *hnRNPL* suppression using 200 pM si-C at 72 h post-electroporation (Supplementary Figure S12A, B). Both *hnRNPL* RNA and protein showed a significant reduction by ∼60% compared with control (Figure 5A–C). Thus, we then applied paired-end sequencing on the polyadenylated transcriptome, performing four biological replicates for each treatment (see the Materials and Methods). RNA-seq analysis confirmed proper clustering of the samples (principal component analysis; Supplementary Figure S12C) and a strong, specific reduction of *hnRNPL* transcript with other hnRNPs unaltered by the treatment (Supplementary Figure S12D). AS analysis [the same tool (SUPPA) and the same parameters (*P*-value <0.05) previously employed] of RNA-seq data from *hnRNPL* knockdown in human hiNPCs revealed 473 AS events, affecting 287 unique genes, partly overlapping those described following *CHD8* suppression. Specifically, 47.7% of genes affected by at least one aberrant AS event following *hnRNPL* suppression overlapped with those presenting altered AS in *CHD8* knockdown (Figure 5D). Notably, once again, AF, the most affected AS subtype by CHD8 suppression, was revealed to be the most recurrent aberrant AS event type after hnRNPL reduction (Figure 5E), while pathways and GO terms associated with ‘nuclear-transcribed mRNA’ and ‘RNA catabolic process’ were recurrently enriched in the lists of genes affected by dysfunctional splicing in both *CHD8* and *hnRNPL* suppression data (Figure 5F). Finally, although with a reduced percentage of gene overlap (20%), AS analysis of data from *hnRNPL* knockdown in unrelated cellular model systems (HepG2 and K562 cells, ENCODE; see the Materials and Methods) still correlated with the splicing defects described following *CHD8* suppression (Supplementary Figure S12E).

## DISCUSSION

Disruption of *CHD8* from *de novo* protein truncating and structural variants is well established as a highly penetrant risk factor for ASD (3,4,7,76). CHD8, initially described as interacting with β-catenin as a negative regulator of WNT signaling (83,89–91), has important roles during nervous system development (14,83). CHD8 directly binds DNA in ∼7000 genomic locations at H3K27ac- and H3K4me3-enriched regions of highly transcriptionally active promoters and enhancers (11,16). However, with both direct and indirect transcriptional effects observed following *CHD8* suppression, its molecular mode of action in ASD remains unclear (11). CHD8 recruits histone KMT2/MLL methyltransferase complexes (18,92), while a cross-talk with the core PRC2 methyltransferase, Ezh2 (12), cannot be excluded. However, as CHD8 associates with elongating RNAPII, its role in transcriptional elongation needs to be taken into account, especially for highly expressed genes that are densely decorated by histone H3K36me3 (84).

In our ASD-relevant, human neuronal progenitor model system, we observed that *CHD8* suppression is prominently associated with a depletion, rather than a gain, in H3K36me3 histone modification. While an effect at enhancers and promoters could not be completely ruled out, stringent statistical criteria to focus on the strongest and most reliable epigenetic changes supported a drastic depletion of H3K36me3, a phenotype validated by independent quantitative approaches. Genes displaying reduction in this histone mark are enriched for ‘constrained’ genes [intolerant to loss-of-function mutations, gnomAD (71)], ‘FMRP targets in brain’ (72), SFARI ASD genes (https://gene.sfari.org/about-gene-scoring/), ‘essential genes’ (73) and genes whose expression peaks early during nervous system development (M2, M3, post-conception weeks 10–12) (6). Thus, CHD8 could facilitate the methyltransferase activity (SETD2/SETD5) leading to H3K36 trimethylation or act as an inhibitor of KDM2B (93). Modulation of H3K36 methylation relates to cell cycle regulation during neuronal development and differentiation. In fact, mutations in *SETD2* have been described in Sotos syndrome, a childhood overgrowth condition with macrocephaly (94), and in an ASD proband, also presenting macrocephaly (5,95), while disruptive mutations in *SETD5*—a newly described H3K36me3 methyltransferase (96)—are associated with ID/ASD (1,76–79) (97) and 3p25.3 microdeletion syndrome (98). Thus, it is possible that *CHD8* suppression, through its ability to modulate H3K36me3 levels, might lead to aberrant development and proliferation as observed in animal models and in *CHD8*-autistic subjects (9,14).

How can CHD8 modulate H3K36me3? From RNA-seq data, *CHD8* suppression does not correlate with a direct reduction in SETD2/SETD5 levels or the up-regulation in H3K36me3 KDM2B methyltransferase (not shown). However, genes bound at their promoters/enhancers by CHD8 specifically present a significant depletion in H3K36me3, thus suggesting a possible direct interplay between the chromodomain and the H3K36 trimethylases. Indeed, *SETD2* and *CHD8* display similar temporal expression patterns during human brain development (data from the BrainSpan Atlas) (9).

Trimethylation of H3K36 demarcates body regions of actively transcribed genes, providing signals for modulating transcription fidelity, mRNA splicing and DNA damage repair (75). Aberrant H3K36me3 reduction in our data is not directly causative of transcriptional differences, and this is coherent with previous findings (99). Rather, reduced H3K36me3 correlates with altered AS. Previous association of H3K36me3 with AS has been reported (100,101). Splicing differences detected in this study as a consequence of *CHD8* suppression are mirrored in different datasets, even in murine models of ASD [P5 cortices and mNPC (14,62)]. Splicing alterations are, in all cases, identified during nervous system development or in neuronal committed progenitors (14,62), with skipped event and alternative 5′, SE-AF, event types among the most sensitive, thus supporting previous finding of a neuronal splicing defect consequent to *Chd8* haploinsufficiency (36). However, while a significant proportion of genes with reduced H3K36me3 enrichment present altered splicing patterns, a significant amount of other aberrantly spliced genes do not present reduced H3K36me3, but binding sites for CHD8. This observation, while supporting a functional link between H3K36me3, CHD8 and splicing regulation, also suggests a H3K36me3-indirect role for CHD8 in AS regulation (Figure 5G). Moreover, motif analysis of RNA sequences adjacent to the aberrant splicing events predicts a relevant contribution by SRSF, hnRNP and ELAV RBP families, well-established regulators of AS, thus suggesting a mode through which CHD8 could operate. Interestingly, genes correlated with ‘RNA splicing’ and ‘mRNA processing’ are among those bound by CHD8 and displaying aberrant AS. This, presently overlooked molecular mechanism warrants further investigations especially in neurodevelopmental syndromes and ASD in particular.

In order to fill this gap, we resorted to MS/MS analysis in hNPCs. Next to a redundant pool of previously identified CHD8 interactors (81–84), our analysis revealed a significant group of nucleoproteins directly implicated in ‘RNA binding’, ‘RNA processing’ and ‘spliceosome’ regulation. Specifically, many hnRNPs (85), already foreseen by the RNA motif analysis of the sequences adjacent to aberrant spliced events, were independently identified with two N-terminal and C-terminal CHD8 antibodies, as well as in mESCs (Figure 4D, E). Intriguingly, recent human genetics studies identified hnRNPs as candidate genes in neurodevelopmental conditions, strongly implicating disruption of this gene's family in altered brain development (102). Among the others, hnRNPL, preferentially bound to CA-rich elements (103), and a regulator of inducible exon skipping (104), was prioritized for validation. CHD8 robustly interacts with hnRNPL possibly through the N-terminal domain as judged by the more efficient pull-down obtained using the CHD8 C-terminal antibody, with no effect of DNase and EtBr treatments (Figure 4F, G). However, because of the impact of RNase A treatment on this binding, a stabilizing contribution by RNA might be proposed for CHD8/hnRNPL association (Figure 5G). Indeed, hnRNPL was previously reported to bind to chromatin—and enhancers in particular—in an RNA-dependent manner (105). Thus, CHD8/hnRNPL association, possibly also recruiting the Mediator complex (106), might stabilize looping of enhancers/promoters, thus supporting transcription initiation. Indeed, hnRNPL was shown to associate with Med23 (107), while other members of the CHD family (CHD1) were previously reported to interact with MED1/23/6 and coordinate pre-initiation complex (PIC) assembly (Figure 5G).

HnRNPL was also previously reported as a subunit of the human KMT3a/Set2 complex, assisting H3K36 trimethylation *in vivo* (40). This interaction occurs through the SETD2–hnRNP interaction domain, deletion of which leads to reduced H3K36me3 deposition (39). Indeed, hnRNPL also robustly interacts with SETD2 in our hNPCs (Figure 4H). Importantly, siRNA-mediated *hnRNPL* transient reduction in our ASD-relevant model system partially mirrored (47.7%) the dysfunctional AS observed following *CHD8* suppression. These observations reinforce the hypothesis that CHD8-driven H3K36me3-dependent AS regulation is, at least in part, ascribable to hnRNPL–CHD8 association. However, by comparing CHD8 and SETD2 MS experiments [our data and (39)], a number of other hnRNP interactors emerge, thus providing additional functional links between SETD2, hnRNPs and CHD8 with elongating RNAPII to be explored and characterized.

In conclusion, thanks to the identification of hnRNPL—but also other hnRNPs—as new CHD8 interactors, here we uncover a new function for CHD8—and possibly extendable to other members of the CHD family—at the cross-talk between chromatin, transcription and splicing machinery regulation. Thus, dissecting the consequences of elongation-coupled H3K36 methylation dysfunction for transcription fidelity, RNA splicing and DNA damage repair (75) will represent the next challenge for understanding chromatin-linked alterations in neurodevelopmental disorders and ASD in particular.

## DATA AVAILABILITY

The authors submitted all datasets to the GEO under the accession number GSE148057. The MS proteomics data have been deposited in the ProteomeXchange Consortium via the PRIDE (108) partner repository with the dataset identifier PXD025739.

## Supplementary Material

gkac1134_Supplemental_Files

## References

[1] De Rubeis S. , He X., Goldberg A.P., Poultney C.S., Samocha K., Cicek A.E., Kou Y., Liu L., Fromer M., Walker S. et al. Synaptic, transcriptional and chromatin genes disrupted in autism. Nature. 2014; 515:209–215. 25363760 10.1038/nature13772 PMC4402723

[2] Iossifov I. , O’Roak B.J., Sanders S.J., Ronemus M., Krumm N., Levy D., Stessman H.A., Witherspoon K.T., Vives L., Patterson K.E. et al. The contribution of de novo coding mutations to autism spectrum disorder. Nature. 2014; 515:216–221. 25363768 10.1038/nature13908 PMC4313871

[3] Neale B.M. , Kou Y., Liu L., Ma’ayan A., Samocha K.E., Sabo A., Lin C.F., Stevens C., Wang L.S., Makarov V. et al. Patterns and rates of exonic de novo mutations in autism spectrum disorders. Nature. 2012; 485:242–245. 22495311 10.1038/nature11011 PMC3613847

[4] O’Roak B.J. , Deriziotis P., Lee C., Vives L., Schwartz J.J., Girirajan S., Karakoc E., Mackenzie A.P., Ng S.B., Baker C. et al. Exome sequencing in sporadic autism spectrum disorders identifies severe de novo mutations. Nat. Genet. 2011; 43:585–589. 21572417 10.1038/ng.835 PMC3115696

[5] O’Roak B.J. , Vives L., Fu W., Egertson J.D., Stanaway I.B., Phelps I.G., Carvill G., Kumar A., Lee C., Ankenman K. et al. Multiplex targeted sequencing identifies recurrently mutated genes in autism spectrum disorders. Science. 2012; 338:1619–1622. 23160955 10.1126/science.1227764 PMC3528801

[6] Parikshak N.N. , Luo R., Zhang A., Won H., Lowe J.K., Chandran V., Horvath S., Geschwind D.H. Integrative functional genomic analyses implicate specific molecular pathways and circuits in autism. Cell. 2013; 155:1008–1021. 24267887 10.1016/j.cell.2013.10.031 PMC3934107

[7] Talkowski M.E. , Rosenfeld J.A., Blumenthal I., Pillalamarri V., Chiang C., Heilbut A., Ernst C., Hanscom C., Rossin E., Lindgren A.M. et al. Sequencing chromosomal abnormalities reveals neurodevelopmental loci that confer risk across diagnostic boundaries. Cell. 2012; 149:525–537. 22521361 10.1016/j.cell.2012.03.028 PMC3340505

[8] Abrahams B.S. , Arking D.E., Campbell D.B., Mefford H.C., Morrow E.M., Weiss L.A., Menashe I., Wadkins T., Banerjee-Basu S., Packer A. SFARI gene 2.0: a community-driven knowledgebase for the autism spectrum disorders (ASDs). Mol. Autism. 2013; 4:36. 24090431 10.1186/2040-2392-4-36 PMC3851189

[9] Bernier R. , Golzio C., Xiong B., Stessman H.A., Coe B.P., Penn O., Witherspoon K., Gerdts J., Baker C., Vulto-van Silfhout A.T. et al. Disruptive CHD8 mutations define a subtype of autism early in development. Cell. 2014; 158:263–276. 24998929 10.1016/j.cell.2014.06.017 PMC4136921

[10] Yasin H. , Gibson W.T., Langlois S., Stowe R.M., Tsang E.S., Lee L., Poon J., Tran G., Tyson C., Wong C.K. et al. A distinct neurodevelopmental syndrome with intellectual disability, autism spectrum disorder, characteristic facies, and macrocephaly is caused by defects in CHD8. J. Hum. Genet. 2019; 64:271–280. 30670789 10.1038/s10038-019-0561-0

[11] Sugathan A. , Biagioli M., Golzio C., Erdin S., Blumenthal I., Manavalan P., Ragavendran A., Brand H., Lucente D., Miles J. et al. CHD8 regulates neurodevelopmental pathways associated with autism spectrum disorder in neural progenitors. Proc. Natl Acad. Sci. USA. 2014; 111:E4468–E4477. 25294932 10.1073/pnas.1405266111 PMC4210312

[12] Durak O. , Gao F., Kaeser-Woo Y.J., Rueda R., Martorell A.J., Nott A., Liu C.Y., Watson L.A., Tsai L.H. Chd8 mediates cortical neurogenesis via transcriptional regulation of cell cycle and wnt signaling. Nat. Neurosci. 2016; 19:1477–1488. 27694995 10.1038/nn.4400 PMC5386887

[13] Katayama Y. , Nishiyama M., Shoji H., Ohkawa Y., Kawamura A., Sato T., Suyama M., Takumi T., Miyakawa T., Nakayama K.I. CHD8 haploinsufficiency results in autistic-like phenotypes in mice. Nature. 2016; 537:675–679. 27602517 10.1038/nature19357

[14] Suetterlin P. , Hurley S., Mohan C., Riegman K.L.H., Pagani M., Caruso A., Ellegood J., Galbusera A., Crespo-Enriquez I., Michetti C. et al. Altered neocortical gene expression, brain overgrowth and functional over-connectivity in chd8 haploinsufficient mice. Cereb. Cortex. 2018; 28:2192–2206. 29668850 10.1093/cercor/bhy058 PMC6018918

[15] Hurley S. , Mohan C., Suetterlin P., Ellingford R., Riegman K.L.H., Ellegood J., Caruso A., Michetti C., Brock O., Evans R. et al. Distinct, dosage-sensitive requirements for the autism-associated factor CHD8 during cortical development. Mol. Autism. 2021; 12:16. 33627187 10.1186/s13229-020-00409-3 PMC7905672

[16] Cotney J. , Muhle R.A., Sanders S.J., Liu L., Willsey A.J., Niu W., Liu W., Klei L., Lei J., Yin J. et al. The autism-associated chromatin modifier CHD8 regulates other autism risk genes during human neurodevelopment. Nat. Commun. 2015; 6:6404. 25752243 10.1038/ncomms7404 PMC4355952

[17] Venkatesh S. , Workman J.L. Histone exchange, chromatin structure and the regulation of transcription. Nat. Rev. Mol. Cell Biol. 2015; 16:178–189. 25650798 10.1038/nrm3941

[18] Thompson B.A. , Tremblay V., Lin G., Bochar D.A. CHD8 is an ATP-dependent chromatin remodeling factor that regulates beta-catenin target genes. Mol. Cell. Biol. 2008; 28:3894–3904. 18378692 10.1128/MCB.00322-08 PMC2423111

[19] Lee Y. , Park D., Iyer V.R. The ATP-dependent chromatin remodeler chd1 is recruited by transcription elongation factors and maintains H3K4me3/H3K36me3 domains at actively transcribed and spliced genes. Nucleic Acids Res. 2017; 45:7180–7190. 28460001 10.1093/nar/gkx321 PMC5499586

[20] Kizer K.O. , Phatnani H.P., Shibata Y., Hall H., Greenleaf A.L., Strahl B.D. A novel domain in set2 mediates RNA polymerase II interaction and couples histone H3 K36 methylation with transcript elongation. Mol. Cell. Biol. 2005; 25:3305–3316. 15798214 10.1128/MCB.25.8.3305-3316.2005 PMC1069628

[21] Krogan N.J. , Kim M., Tong A., Golshani A., Cagney G., Canadien V., Richards D.P., Beattie B.K., Emili A., Boone C. et al. Methylation of histone H3 by set2 in *Saccharomyces cerevisiae* is linked to transcriptional elongation by RNA polymerase II. Mol. Cell. Biol. 2003; 23:4207–4218. 12773564 10.1128/MCB.23.12.4207-4218.2003 PMC427527

[22] Radman-Livaja M. , Quan T.K., Valenzuela L., Armstrong J.A., van Welsem T., Kim T., Lee L.J., Buratowski S., van Leeuwen F., Rando O.J. et al. A key role for chd1 in histone H3 dynamics at the 3′ ends of long genes in yeast. PLoS Genet. 2012; 8:e1002811. 22807688 10.1371/journal.pgen.1002811 PMC3395613

[23] Smolle M. , Venkatesh S., Gogol M.M., Li H., Zhang Y., Florens L., Washburn M.P., Workman J.L. Chromatin remodelers isw1 and chd1 maintain chromatin structure during transcription by preventing histone exchange. Nat. Struct. Mol. Biol. 2012; 19:884–892. 22922743 10.1038/nsmb.2312 PMC3560298

[24] De Conti L. , Baralle M., Buratti E. Exon and intron definition in pre-mRNA splicing. Wiley Interdiscipl. Rev. RNA. 2013; 4:49–60. 10.1002/wrna.1140 23044818

[25] Lee Y. , Rio D.C. Mechanisms and regulation of alternative pre-mRNA splicing. Annu. Rev. Biochem. 2015; 84:291–323. 25784052 10.1146/annurev-biochem-060614-034316 PMC4526142

[26] Witten J.T. , Ule J. Understanding splicing regulation through RNA splicing maps. Trends Genet. 2011; 27:89–97. 21232811 10.1016/j.tig.2010.12.001 PMC3165201

[27] Ip J.Y. , Schmidt D., Pan Q., Ramani A.K., Fraser A.G., Odom D.T., Blencowe B.J. Global impact of RNA polymerase II elongation inhibition on alternative splicing regulation. Genome Res. 2011; 21:390–401. 21163941 10.1101/gr.111070.110 PMC3044853

[28] Kornblihtt A.R. Coupling transcription and alternative splicing. Adv. Exp. Med. Biol. 2007; 623:175–189. 18380347 10.1007/978-0-387-77374-2_11

[29] Kornblihtt A.R. , Schor I.E., Allo M., Blencowe B.J. When chromatin meets splicing. Nat. Struct. Mol. Biol. 2009; 16:902–903. 19739285 10.1038/nsmb0909-902

[30] Luco R.F. , Misteli T. More than a splicing code: integrating the role of RNA, chromatin and non-coding RNA in alternative splicing regulation. Curr. Opin. Genet. Dev. 2011; 21:366–372. 21497503 10.1016/j.gde.2011.03.004 PMC6317717

[31] Luco R.F. , Pan Q., Tominaga K., Blencowe B.J., Pereira-Smith O.M., Misteli T. Regulation of alternative splicing by histone modifications. Science. 2010; 327:996–1000. 20133523 10.1126/science.1184208 PMC2913848

[32] Naftelberg S. , Schor I.E., Ast G., Kornblihtt A.R. Regulation of alternative splicing through coupling with transcription and chromatin structure. Annu. Rev. Biochem. 2015; 84:165–198. 26034889 10.1146/annurev-biochem-060614-034242

[33] Nilsen T.W. , Graveley B.R. Expansion of the eukaryotic proteome by alternative splicing. Nature. 2010; 463:457–463. 20110989 10.1038/nature08909 PMC3443858

[34] Saldi T. , Cortazar M.A., Sheridan R.M., Bentley D.L. Coupling of RNA polymerase II transcription elongation with pre-mRNA splicing. J. Mol. Biol. 2016; 428:2623–2635. 27107644 10.1016/j.jmb.2016.04.017 PMC4893998

[35] Cuajungco M.P. , Leyne M., Mull J., Gill S.P., Lu W., Zagzag D., Axelrod F.B., Maayan C., Gusella J.F., Slaugenhaupt S.A. Tissue-specific reduction in splicing efficiency of IKBKAP due to the major mutation associated with familial dysautonomia. Am. J. Hum. Genet. 2003; 72:749–758. 12577200 10.1086/368263 PMC1180251

[36] Gompers A.L. , Su-Feher L., Ellegood J., Copping N.A., Riyadh M.A., Stradleigh T.W., Pride M.C., Schaffler M.D., Wade A.A., Catta-Preta R. et al. Germline chd8 haploinsufficiency alters brain development in mouse. Nat. Neurosci. 2017; 20:1062–1073. 28671691 10.1038/nn.4592 PMC6008102

[37] Mordes D. , Luo X., Kar A., Kuo D., Xu L., Fushimi K., Yu G., Sternberg P. Jr, Wu J.Y Pre-mRNA splicing and retinitis pigmentosa. Mol. Vis. 2006; 12:1259–1271. 17110909 PMC2683577

[38] Wang E.T. , Cody N.A., Jog S., Biancolella M., Wang T.T., Treacy D.J., Luo S., Schroth G.P., Housman D.E., Reddy S. et al. Transcriptome-wide regulation of pre-mRNA splicing and mRNA localization by muscleblind proteins. Cell. 2012; 150:710–724. 22901804 10.1016/j.cell.2012.06.041 PMC3428802

[39] Bhattacharya S. , Levy M.J., Zhang N., Li H., Florens L., Washburn M.P., Workman J.L. The methyltransferase SETD2 couples transcription and splicing by engaging mRNA processing factors through its SHI domain. Nat. Commun. 2021; 12:1443. 33664260 10.1038/s41467-021-21663-w PMC7933334

[40] Yuan W. , Xie J., Long C., Erdjument-Bromage H., Ding X., Zheng Y., Tempst P., Chen S., Zhu B., Reinberg D Heterogeneous nuclear ribonucleoprotein l is a subunit of human KMT3a/Set2 complex required for H3 lys-36 trimethylation activity in vivo. J. Biol. Chem. 2009; 284:15701–15707. 19332550 10.1074/jbc.M808431200 PMC2708867

[41] Sheridan S.D. , Theriault K.M., Reis S.A., Zhou F., Madison J.M., Daheron L., Loring J.F., Haggarty S.J. Epigenetic characterization of the FMR1 gene and aberrant neurodevelopment in human induced pluripotent stem cell models of fragile X syndrome. PLoS One. 2011; 6:e26203. 22022567 10.1371/journal.pone.0026203 PMC3192166

[42] Bernstein B.E. , Mikkelsen T.S., Xie X., Kamal M., Huebert D.J., Cuff J., Fry B., Meissner A., Wernig M., Plath K. et al. A bivalent chromatin structure marks key developmental genes in embryonic stem cells. Cell. 2006; 125:315–326. 16630819 10.1016/j.cell.2006.02.041

[43] Li H. , Durbin R. Fast and accurate short read alignment with Burrows–Wheeler transform. Bioinformatics. 2009; 25:1754–1760. 19451168 10.1093/bioinformatics/btp324 PMC2705234

[44] 1000 Genome Project Data Processing Subgroup Li H. , Handsaker B., Wysoker A., Fennell T., Ruan J., Homer N., Marth G., Abecasis G., Durbin R. The sequence alignment/map format and SAMtools. Bioinformatics. 2009; 25:2078–2079. 19505943 10.1093/bioinformatics/btp352 PMC2723002

[45] Zhang Y. , Liu T., Meyer C.A., Eeckhoute J., Johnson D.S., Bernstein B.E., Nusbaum C., Myers R.M., Brown M., Li W. et al. Model-based analysis of chip-Seq (MACS). Genome Biol. 2008; 9:R137. 18798982 10.1186/gb-2008-9-9-r137 PMC2592715

[46] Frankish A. , Diekhans M., Ferreira A.M., Johnson R., Jungreis I., Loveland J., Mudge J.M., Sisu C., Wright J., Armstrong J. et al. GENCODE reference annotation for the human and mouse genomes. Nucleic Acids Res. 2019; 47:D766–D773. 30357393 10.1093/nar/gky955 PMC6323946

[47] Yu G. , Wang L.G., Han Y., He Q.Y. clusterProfiler: an r package for comparing biological themes among gene clusters. Omics. 2012; 16:284–287. 22455463 10.1089/omi.2011.0118 PMC3339379

[48] Ernst J. , Kellis M. ChromHMM: automating chromatin-state discovery and characterization. Nat. Methods. 2012; 9:215–216. 22373907 10.1038/nmeth.1906 PMC3577932

[49] Quinlan A.R. , Hall I.M. BEDTools: a flexible suite of utilities for comparing genomic features. Bioinformatics. 2010; 26:841–842. 20110278 10.1093/bioinformatics/btq033 PMC2832824

[50] Stark R. , Brown G. DiffBind: differential binding analysis of chipseq peak data. Bioconductor 3.16. 2011; 10.18129/B9.bioc.DiffBind.

[51] Ross-Innes C.S. , Stark R., Teschendorff A.E., Holmes K.A., Ali H.R., Dunning M.J., Brown G.D., Gojis O., Ellis I.O., Green A.R. et al. Differential oestrogen receptor binding is associated with clinical outcome in breast cancer. Nature. 2012; 481:389–393. 22217937 10.1038/nature10730 PMC3272464

[52] Wickham H. ggplot2 Elegant Graphics for Data Analysis. 2009; NY Springer-Verlag.

[53] Ramirez F. , Ryan D.P., Gruning B., Bhardwaj V., Kilpert F., Richter A.S., Heyne S., Dundar F., Manke T. deepTools2: a next generation web server for deep-sequencing data analysis. Nucleic Acids Res. 2016; 44:W160–W165. 27079975 10.1093/nar/gkw257 PMC4987876

[54] Diaz A. , Park K., Lim D.A., Song J.S. Normalization, bias correction, and peak calling for chip-seq. Stat. Appl. Genet. Mol. Biol. 2012; 11:Article 9. 10.1515/1544-6115.1750 PMC3342857 22499706

[55] Gibbons R. , Hedeker D., Davis J. Estimation of effect size from a series of experiments involving paired comparisons. J. Educ. Stat. 1993; 18:271–279.

[56] Robinson J.T. , Thorvaldsdottir H., Winckler W., Guttman M., Lander E.S., Getz G., Mesirov J.P. Integrative genomics viewer. Nat. Biotechnol. 2011; 29:24–26. 21221095 10.1038/nbt.1754 PMC3346182

[57] Bray N.L. , Pimentel H., Melsted P., Pachter L. Near-optimal probabilistic RNA-seq quantification. Nat. Biotechnol. 2016; 34:525–527. 27043002 10.1038/nbt.3519

[58] Trincado J.L. , Entizne J.C., Hysenaj G., Singh B., Skalic M., Elliott D.J., Eyras E. SUPPA2: fast, accurate, and uncertainty-aware differential splicing analysis across multiple conditions. Genome Biol. 2018; 19:40. 29571299 10.1186/s13059-018-1417-1 PMC5866513

[59] Dobin A. , Davis C.A., Schlesinger F., Drenkow J., Zaleski C., Jha S., Batut P., Chaisson M., Gingeras T.R. STAR: ultrafast universal RNA-seq aligner. Bioinformatics. 2013; 29:15–21. 23104886 10.1093/bioinformatics/bts635 PMC3530905

[60] Shen S. , Park J.W., Lu Z.X., Lin L., Henry M.D., Wu Y.N., Zhou Q., Xing Y. rMATS: robust and flexible detection of differential alternative splicing from replicate RNA-Seq data. Proc. Natl Acad. Sci. USA. 2014; 111:E5593–E5601. 25480548 10.1073/pnas.1419161111 PMC4280593

[61] Garrido-Martin D. , Palumbo E., Guigo R., Breschi A. ggsashimi: sashimi plot revised for browser- and annotation-independent splicing visualization. PLoS Comput. Biol. 2018; 14:e1006360. 30118475 10.1371/journal.pcbi.1006360 PMC6114895

[62] Sood S. , Weber C.M., Hodges H.C., Krokhotin A., Shalizi A., Crabtree G.R. CHD8 dosage regulates transcription in pluripotency and early murine neural differentiation. Proc. Natl Acad. Sci. USA. 2020; 117:22331–22340. 32839322 10.1073/pnas.1921963117 PMC7486765

[63] Smedley D. , Haider S., Ballester B., Holland R., London D., Thorisson G., Kasprzyk A. BioMart—biological queries made easy. BMC Genomics. 2009; 10:22. 19144180 10.1186/1471-2164-10-22 PMC2649164

[64] Ray D. , Kazan H., Cook K.B., Weirauch M.T., Najafabadi H.S., Li X., Gueroussov S., Albu M., Zheng H., Yang A. et al. A compendium of RNA-binding motifs for decoding gene regulation. Nature. 2013; 499:172–177. 23846655 10.1038/nature12311 PMC3929597

[65] Cock P.J. , Antao T., Chang J.T., Chapman B.A., Cox C.J., Dalke A., Friedberg I., Hamelryck T., Kauff F., Wilczynski B. et al. Biopython: freely available python tools for computational molecular biology and bioinformatics. Bioinformatics. 2009; 25:1422–1423. 19304878 10.1093/bioinformatics/btp163 PMC2682512

[66] Durinck S. , Spellman P.T., Birney E., Huber W. Mapping identifiers for the integration of genomic datasets with the R/Bioconductor package biomaRt. Nat. Protocols. 2009; 4:1184–1191. 19617889 10.1038/nprot.2009.97 PMC3159387

[67] Gagnon K.T. , Li L., Janowski B.A., Corey D.R. Analysis of nuclear RNA interference in human cells by subcellular fractionation and argonaute loading. Nat. Protoc. 2014; 9:2045–2060. 25079428 10.1038/nprot.2014.135 PMC4251768

[68] Aguilan J.T. , Kulej K., Sidoli S. Guide for protein fold change and p-value calculation for non-experts in proteomics. Mol. Omics. 2020; 16:573–582. 32968743 10.1039/d0mo00087f

[69] ENCODE Project Consortium An integrated encyclopedia of DNA elements in the human genome. Nature. 2012; 489:57–74. 22955616 10.1038/nature11247 PMC3439153

[70] Davis C.A. , Hitz B.C., Sloan C.A., Chan E.T., Davidson J.M., Gabdank I., Hilton J.A., Jain K., Baymuradov U.K., Narayanan A.K. et al. The encyclopedia of DNA elements (ENCODE): data portal update. Nucleic Acids Res. 2018; 46:D794–D801. 29126249 10.1093/nar/gkx1081 PMC5753278

[71] Karczewski K.J. , Francioli L.C., Tiao G., Cummings B.B., Alfoldi J., Wang Q., Collins R.L., Laricchia K.M., Ganna A., Birnbaum D.P. et al. The mutational constraint spectrum quantified from variation in 141,456 humans. Nature. 2020; 581:434–443. 32461654 10.1038/s41586-020-2308-7 PMC7334197

[72] Darnell J.C. , Van Driesche S.J., Zhang C., Hung K.Y., Mele A., Fraser C.E., Stone E.F., Chen C., Fak J.J., Chi S.W. et al. FMRP stalls ribosomal translocation on mRNAs linked to synaptic function and autism. Cell. 2011; 146:247–261. 21784246 10.1016/j.cell.2011.06.013 PMC3232425

[73] Wang T. , Birsoy K., Hughes N.W., Krupczak K.M., Post Y., Wei J.J., Lander E.S., Sabatini D.M. Identification and characterization of essential genes in the human genome. Science. 2015; 350:1096–1101. 26472758 10.1126/science.aac7041 PMC4662922

[74] Wade A.A. , Lim K., Catta-Preta R., Nord A.S. Common CHD8 genomic targets contrast with model-specific transcriptional impacts of CHD8 haploinsufficiency. Front. Mol. Neurosci. 2018; 11:481. 30692911 10.3389/fnmol.2018.00481 PMC6339895

[75] Wagner E.J. , Carpenter P.B. Understanding the language of lys36 methylation at histone H3. Nat. Rev. Mol. Cell Biol. 2012; 13:115–126. 22266761 10.1038/nrm3274 PMC3969746

[76] Satterstrom F.K. , Kosmicki J.A., Wang J., Breen M.S., De Rubeis S., An J.Y., Peng M., Collins R., Grove J., Klei L. et al. Large-scale exome sequencing study implicates both developmental and functional changes in the neurobiology of autism. Cell. 2020; 180:568–584. 31981491 10.1016/j.cell.2019.12.036 PMC7250485

[77] Koh T.W. , Verstreken P., Bellen H.J. Dap160/intersectin acts as a stabilizing scaffold required for synaptic development and vesicle endocytosis. Neuron. 2004; 43:193–205. 15260956 10.1016/j.neuron.2004.06.029

[78] Pechstein A. , Shupliakov O., Haucke V. Intersectin 1: a versatile actor in the synaptic vesicle cycle. Biochem. Soc. Trans. 2010; 38:181–186. 20074056 10.1042/BST0380181

[79] Sengar A.S. , Ellegood J., Yiu A.P., Wang H., Wang W., Juneja S.C., Lerch J.P., Josselyn S.A., Henkelman R.M., Salter M.W. et al. Vertebrate intersectin1 is repurposed to facilitate cortical midline connectivity and higher order cognition. J. Neurosci. 2013; 33:4055–4065. 23447614 10.1523/JNEUROSCI.4428-12.2013 PMC6619305

[80] Jakob B. , Kochlamazashvili G., Japel M., Gauhar A., Bock H.H., Maritzen T., Haucke V. Intersectin 1 is a component of the reelin pathway to regulate neuronal migration and synaptic plasticity in the hippocampus. Proc. Natl Acad. Sci. USA. 2017; 114:5533–5538. 28484035 10.1073/pnas.1704447114 PMC5448185

[81] Batsukh T. , Pieper L., Koszucka A.M., von Velsen N., Hoyer-Fender S., Elbracht M., Bergman J.E., Hoefsloot L.H., Pauli S. CHD8 interacts with CHD7, a protein which is mutated in CHARGE syndrome. Hum. Mol. Genet. 2010; 19:2858–2866. 20453063 10.1093/hmg/ddq189

[82] Cerase A. , Young A.N., Ruiz N.B., Buness A., Sant G.M., Arnold M., Di Giacomo M., Ascolani M., Kumar M., Hierholzer A. et al. Chd8 regulates X chromosome inactivation in mouse through fine-tuning control of Xist expression. Commun. Biol. 2021; 4:485. 33859315 10.1038/s42003-021-01945-1 PMC8050208

[83] Nishiyama M. , Oshikawa K., Tsukada Y., Nakagawa T., Iemura S., Natsume T., Fan Y., Kikuchi A., Skoultchi A.I., Nakayama K.I. CHD8 suppresses p53-mediated apoptosis through histone H1 recruitment during early embryogenesis. Nat. Cell Biol. 2009; 11:172–182. 19151705 10.1038/ncb1831 PMC3132516

[84] Rodriguez-Paredes M. , Ceballos-Chavez M., Esteller M., Garcia-Dominguez M., Reyes J.C. The chromatin remodeling factor CHD8 interacts with elongating RNA polymerase II and controls expression of the cyclin E2 gene. Nucleic Acids Res. 2009; 37:2449–2460. 19255092 10.1093/nar/gkp101 PMC2677868

[85] Geuens T. , Bouhy D., Timmerman V. The hnRNP family: insights into their role in health and disease. Hum. Genet. 2016; 135:851–867. 27215579 10.1007/s00439-016-1683-5 PMC4947485

[86] Heiner M. , Hui J., Schreiner S., Hung L.H., Bindereif A. HnRNP L-mediated regulation of mammalian alternative splicing by interference with splice site recognition. RNA Biol. 2010; 7:56–64. 19946215 10.4161/rna.7.1.10402

[87] Rossbach O. , Hung L.H., Khrameeva E., Schreiner S., Konig J., Curk T., Zupan B., Ule J., Gelfand M.S., Bindereif A. Crosslinking-immunoprecipitation (iCLIP) analysis reveals global regulatory roles of hnRNP L. RNA Biol. 2014; 11:146–155. 24526010 10.4161/rna.27991 PMC3973733

[88] McClory S.P. , Lynch K.W., Ling J.P. HnRNP l represses cryptic exons. RNA. 2018; 24:761–768. 29581412 10.1261/rna.065508.117 PMC5959245

[89] Nishiyama M. , Nakayama K., Tsunematsu R., Tsukiyama T., Kikuchi A., Nakayama K.I. Early embryonic death in mice lacking the beta-catenin-binding protein duplin. Mol. Cell. Biol. 2004; 24:8386–8394. 15367660 10.1128/MCB.24.19.8386-8394.2004 PMC516734

[90] Nishiyama M. , Skoultchi A.I., Nakayama K.I. Histone H1 recruitment by CHD8 is essential for suppression of the Wnt-beta-catenin signaling pathway. Mol. Cell. Biol. 2012; 32:501–512. 22083958 10.1128/MCB.06409-11 PMC3255766

[91] Sakamoto I. , Kishida S., Fukui A., Kishida M., Yamamoto H., Hino S., Michiue T., Takada S., Asashima M., Kikuchi A. A novel beta-catenin-binding protein inhibits beta-catenin-dependent Tcf activation and axis formation. J. Biol. Chem. 2000; 275:32871–32878. 10921920 10.1074/jbc.M004089200

[92] Zhao C. , Dong C., Frah M., Deng Y., Marie C., Zhang F., Xu L., Ma Z., Dong X., Lin Y. et al. Dual requirement of CHD8 for chromatin landscape establishment and histone methyltransferase recruitment to promote CNS myelination and repair. Dev. Cell. 2018; 45:753–768. 29920279 10.1016/j.devcel.2018.05.022 PMC6063525

[93] He J. , Shen L., Wan M., Taranova O., Wu H., Zhang Y. Kdm2b maintains murine embryonic stem cell status by recruiting PRC1 complex to CpG islands of developmental genes. Nat. Cell Biol. 2013; 15:373–384. 23502314 10.1038/ncb2702 PMC4078788

[94] Luscan A. , Laurendeau I., Malan V., Francannet C., Odent S., Giuliano F., Lacombe D., Touraine R., Vidaud M., Pasmant E. et al. Mutations in SETD2 cause a novel overgrowth condition. J. Med. Genet. 2014; 51:512–517. 24852293 10.1136/jmedgenet-2014-102402

[95] Lumish H.S. , Wynn J., Devinsky O., Chung W.K. Brief report: SETD2 mutation in a child with autism, intellectual disabilities and epilepsy. J. Autism Dev. Disord. 2015; 45:3764–3770. 26084711 10.1007/s10803-015-2484-8

[96] Sessa A. , Fagnocchi L., Mastrototaro G., Massimino L., Zaghi M., Indrigo M., Cattaneo S., Martini D., Gabellini C., Pucci C. et al. SETD5 regulates chromatin methylation state and preserves global transcriptional fidelity during brain development and neuronal wiring. Neuron. 2019; 104:271–289. 31515109 10.1016/j.neuron.2019.07.013

[97] Rawlins L.E. , Stals K.L., Eason J.D., Turnpenny P.D. De novo SETD5 nonsense mutation associated with diaphragmatic hernia and severe cerebral cortical dysplasia. Clin. Dysmorphol. 2017; 26:95–97. 28263952 10.1097/MCD.0000000000000144

[98] Kellogg G. , Sum J., Wallerstein R. Deletion of 3p25.3 in a patient with intellectual disability and dysmorphic features with further definition of a critical region. Am. J. Med. Genet. A. 2013; 161:1405–1408. 10.1002/ajmg.a.35876 23613140

[99] Simon J.M. , Hacker K.E., Singh D., Brannon A.R., Parker J.S., Weiser M., Ho T.H., Kuan P.F., Jonasch E., Furey T.S. et al. Variation in chromatin accessibility in human kidney cancer links H3K36 methyltransferase loss with widespread RNA processing defects. Genome Res. 2014; 24:241–250. 24158655 10.1101/gr.158253.113 PMC3912414

[100] de Almeida S.F. , Grosso A.R., Koch F., Fenouil R., Carvalho S., Andrade J., Levezinho H., Gut M., Eick D., Gut I. et al. Splicing enhances recruitment of methyltransferase HYPB/Setd2 and methylation of histone H3 Lys36. Nat. Struct. Mol. Biol. 2011; 18:977–983. 21792193 10.1038/nsmb.2123

[101] Kim S. , Kim H., Fong N., Erickson B., Bentley D.L. Pre-mRNA splicing is a determinant of histone H3K36 methylation. Proc. Natl Acad, Sci. USA. 2011; 108:13564–13569. 21807997 10.1073/pnas.1109475108 PMC3158196

[102] Gillentine M.A. , Wang T., Hoekzema K., Rosenfeld J., Liu P., Guo H., Kim C.N., De Vries B.B.A., Vissers L., Nordenskjold M. et al. Rare deleterious mutations of HNRNP genes result in shared neurodevelopmental disorders. Genome Med. 2021; 13:63. 33874999 10.1186/s13073-021-00870-6 PMC8056596

[103] Han S.P. , Tang Y.H., Smith R. Functional diversity of the hnRNPs: past, present and perspectives. Biochem. J. 2010; 430:379–392. 20795951 10.1042/BJ20100396

[104] Melton A.A. , Jackson J., Wang J., Lynch K.W. Combinatorial control of signal-induced exon repression by hnRNP l and PSF. Mol. Cell. Biol. 2007; 27:6972–6984. 17664280 10.1128/MCB.00419-07 PMC2099238

[105] Zhao Y. , Zhou J., He L., Li Y., Yuan J., Sun K., Chen X., Bao X., Esteban M.A., Sun H. et al. MyoD induced enhancer RNA interacts with hnRNPL to activate target gene transcription during myogenic differentiation. Nat. Commun. 2019; 10:5787. 31857580 10.1038/s41467-019-13598-0 PMC6923398

[106] Quevedo M. , Meert L., Dekker M.R., Dekkers D.H.W., Brandsma J.H., van den Berg D.L.C., Ozgur Z., van I.W.F.J., Demmers J., Fornerod M. et al. Mediator complex interaction partners organize the transcriptional network that defines neural stem cells. Nat. Commun. 2019; 10:2669. 31209209 10.1038/s41467-019-10502-8 PMC6573065

[107] Huang Y. , Li W., Yao X., Lin Q.J., Yin J.W., Liang Y., Heiner M., Tian B., Hui J., Wang G. Mediator complex regulates alternative mRNA processing via the MED23 subunit. Mol. Cell. 2012; 45:459–469. 22264826 10.1016/j.molcel.2011.12.022 PMC3288850

[108] Perez-Riverol Y. , Csordas A., Bai J., Bernal-Llinares M., Hewapathirana S., Kundu D.J., Inuganti A., Griss J., Mayer G., Eisenacher M. et al. The PRIDE database and related tools and resources in 2019: improving support for quantification data. Nucleic Acids Res. 2019; 47:D442–D450. 30395289 10.1093/nar/gky1106 PMC6323896

