# Supplementary material for: CHD8 suppression impacts on histone H3 lysine 36 trimethylation and alters RNA alternative splicing
Source: Nucleic Acids Res. 2022 Dec 20;50(22):12809–28. doi: 10.1093/nar/gkac1134 (PMC9825192; doi:10.1093/nar/gkac1134)
Supplement: gkac1134_Supplemental_Files [file gkac1134_supplemental_files.zip › Legends to Supplementary_Table_1_2022_11_03.docx]

*Suppl. Table 1 Capture*

*Fig.1G_gene_lists*. Description and references for ‘ASD’, ‘neurodevelopment’, ‘co-expression modules in brain’ and ‘intolerance to loss of function’ gene lists. These data were used for the gene set enrichment analysis presented in Fig. 1G for genes losing H3K36me3 enrichment following *CHD8* knock-down.

*Fig.1G_enrichment*. Results of the gene set enrichment analysis for genes losing H3K36me3 enrichment following *CHD8* knock-down as presented in Fig. 1G.

*Fig.2H_BP_enrichment.* Full results of the Gene Ontology Biological Process (GO BP) enrichment shown in Fig. 2H for genes belonging to Cluster #1 and #2, with high/medium CHD8 binding enrichment in control hiNPCs and a lower H3K36me3 enrichment in *CHD8* knock-down.

*Fig.3E_H3K36me3_lost.* Full results of the Gene Ontology Biological Process (GO BP) and KEGG pathways enrichment shown in Fig. 3E top panel for genes losing H3K36me3 peaks following *CHD8* knock-down.

*Fig.3E_CHD8_bound.* Full results of the Gene Ontology Biological Process (GO BP) and KEGG pathways enrichment presented in Fig. 3E bottom panel for genes bound by CHD8.

*Fig.3G-I_PCR_qPCR_Primers.* Complete list of primers used for semi quantitative and quantitative PCR experiments. Top part primers list used for semiquantitative PCR for AS validation of Fig.3G-H, bottom part primers list used for ChIP-qPCR in Fig.3I.

*Fig.4C-E_IP_Proteins_List.* Complete list of CHD8 interacting proteins identified by MS/MS, immunoprecipitating CHD8 with Ab #17 (see Fig.4C/D, left part), Ab #18 (see Fig.4C/D, right part) or intersection of peptides obtained using both antibodies as reported in Fig.4D-E. Only statistically significant proteins with positive fold change from three biological replicates were reported (A+B+C).

*Fig. 5F_GO terms hnRNPL_CHD8.* Full list of the Gene Ontology (GO) enrichment presented in Fig. 5F for genes presenting aberrant splicing following CHD8 and hnRNPL suppression (intersection in Fig. 5D).

*Supp.Fig.8_BP_enrichment.* Full results of the Gene Ontology Biological Process (GO BP) enrichment showed in Suppl. Fig. 8 for genes belonging to Cluster #3 (see Fig. 2G), with low levels of CHD8 binding enrichment in control hiNPCs and a lower H3K36me3 enrichment in *CHD8* knock-down.

*Supp.Fig.10G.* Full results of the Gene Ontology Biological Process (GO BP) enrichment showed in Suppl. Fig. 10G for genes presenting aberrant alternative splicing in iPS-derived NPC with reduced levels of *CHD8*.

*Supp.Fig.10H.* Full results of the Gene Ontology Biological Process (GO BP) enrichment showed in Suppl. Fig. 10H for genes presenting aberrant alternative splicing in mouse P5 cortex with reduced levels of *Chd8*.

*Supp.Fig.10I.* Full results of the Gene Ontology Biological Process (GO BP) enrichment showed in Suppl. Fig. 10I for genes presenting aberrant alternative splicing in mNPCs with reduced levels of *Chd8*.

*Supp.Fig.11B-C_IP_Protein_List.* Full list of CHD8 interacting proteins identified by MS/MS immunoprecipitating CHD8 with Ab #17 or Ab #18. Only statistically significant proteins with positive fold change were reported for each biological replicate separately (A or B or C) as depicted in Supp.Fig.11B (Ab #17) and Supp.Fig.11C (Ab #18). Each biological replicate was initially analyzed separately. On the right part of the spreadsheet, the list of CHD8 interacting proteins obtained from the overlap between the 3 biological replicates with each antibody (Ab #17 and Ab #1, respectively) was presented.

*Supp.Fig.11D_IP_overlap_GO_KEGG*. Full results of the Gene Ontology Biological Process (GO BP) and KEGG enrichment of CHD8 interacting proteins, as resulting from the overlap between IP with Ab #17 (A+B+C) and Ab #18 (A+B+C), as presented in Fig.4D/E.

*Supp.Fig.11E_IP_Protein_List.* Full list of CHD8 interacting proteins identified by MS/MS immunoprecipitating CHD8 in hiNPC using Ab #17 and analyzing together two biological replicates (A+B) (right column). Full list of Chd8 interacting proteins identified by MS in mESC was reported on the central columns. Only statistically significant proteins with positive fold change were reported (Supp.Fig.11E). On the right part of the spreadsheet, the list of CHD8/Chd8 interacting proteins emerging from the overlap between these two lists was presented in column K.

*Supp.Fig.11F_IP_overlap_GO_KEGG.* Full results of the Gene Ontology Biological Process (GO BP) and KEGG enrichment of CHD8 interacting proteins, as resulting from the overlap between IP with Ab #17 (A+B) in hiNPC and IP of Chd8 with Bethyl Ab in mESC as reported in Supp.Fig.11F.
