# Supplementary material for: CHD8 suppression impacts on histone H3 lysine 36 trimethylation and alters RNA alternative splicing
Source: Nucleic Acids Res. 2022 Dec 20;50(22):12809–28. doi: 10.1093/nar/gkac1134 (PMC9825192; doi:10.1093/nar/gkac1134)

Supplementary Methods

Reads obtained from ChIP-seq libraries were checked for quality using FastQC (https://www.bioinformatics.babraham.ac.uk/projects/fastqc/) and MultiQC (http://multiqc.info/). The metagene enrichment profile for each histone mark, was normalized against INPUT sample and plotted with deepTools grouping genes in five quintiles according to their level of expression as reported in Sugathan et al. 2014 (1). ChIP-seq samples Spearman's correlation on the enrichment pattern was calculated and plotted with deepTools, version 2.3.5, (2) on all our samples. As a control, the same histone marks ChIP-seq samples in neural progenitor cells (H9 derived) from ENCODE (3,4) were inserted in the correlation plot (ENCSR274OIJ, ENCSR645BCH, ENCSR661MUS, ENCSR449AXO, ENCSR139PIA, ENCSR573CWZ, ENCSR603SVD).

The same approach used for histone marks was carried on for H3K4me1, H3K4me2 and H3K36me3 Sh-*GFP* and Sh2-*CHD8* samples previously sequenced in the Talkowski-Gusella laboratories (Center for Genomic Medicine, Massachusetts General Hospital, Boston, MA, United States).

To check the correlation between histone modifications that mark actively transcribed regions and transcription data, (H3K4me3 and H3K36me3) ChIP-seq and RNA-seq reads were counted by BEDTools multicov, version 2.25.0,(5). They were then normalized by library size and gene length, and displayed in a scatter plot. The LOWESS smoother was computed using R's lowess function and plotted with R.

Supplementary Figures

Figure Suppl. 1 (related to Fig. 1) Quality controls of read mappings and enrichment profiles.

A. The table is showing the quantification of the two main *CHD8* isoforms (*CHD8_201* and *CHD8_212*) in Transcripts Per Kilobase Million (TPM) divided for each Sh-*GFP* and Sh-*CHD8* sample.

B. The bar chart on the left and the representative image on the right report fold change differences in CHD8 protein level comparing control (Sh-*GFP*) and *CHD8* knock-down clones (Sh1-*CHD8*, Sh2-*CHD8*, Sh4-*CHD8*) in western blotting experiments. Down-regulation of CHD8 amount is observed following *CHD8* suppression. Comparable amounts of total protein were loaded and HSP90 was used as loading control. The bars represent normalized CHD8 values relative to Sh-*GFP* controls. Mean values ± s.e. from independent biological replicates (n=4 for Sh4-*CHD8* and n=6 for the other samples) are plotted. T test for two mean population was performed. * $p \leq 0.05$. Levels of CHD8 reduction are indicated in the top part of the panel as fold change (FC) compared to control Sh-GFP.

C. The bar plot represents the total number of raw (light grey), mapped (dark grey) and filtered reads (black) for each condition and histone mark.

D. The bar plot represents the total number of peaks called for each condition and histone mark. Details on peak calling were reported in Materials and Methods.

E. Metagene profiles of the six histone marks analyzed. Different outlines represent five groups of genes sorted based on their RNA expression levels. RNA expression from Sugathan et al. 2014 is calculated in quintiles and represented in shades of grey. Every histone mark displays the expected profile around the Transcription Start Site (TSS) or the gene body [TSS till

Transcription End Site (TES)]. Highly expressed genes (80-100% expression quintiles, light grey line) show enrichment for histone marks associated with active transcription, while histone H3K27me3 shows a corresponding depletion.

F. The heatmap reports the Pearson's correlation values calculated between different samples of this study and ENCODE datasets obtained from human neural progenitor cells [H9 cells ChIP-seq samples (details in Supplementary Methods)]. Correlation values are constructed on ChIP coverage calculated in 10 Kbp bins over the whole genome. Clustering is following the specific histone mark (multiple conditions and ENCODE datasets clusters together), confirming a similar enrichment pattern over the genome. Histone marks presenting enrichment at the transcription start site (H3K4me, H3K27ac) appear to be grouped together (central part of the heatmap).

Figure Suppl. 2 (related to Fig. 1) Peaks comparison across different CHD8 knock-down replicates and independent validation in ChIP-seq samples from Talkowski-Gusella laboratory (Massachusetts General Hospital, Boston, MA, United States.).

A. The bar plots show the total number of peaks called in controls (Sh-*GFP*, Sh-*GFP2*, dark grey) and *CHD8* knock-down (Sh1-*CHD8*, Sh2-*CHD8*, Sh1-*CHD8*, white) for each of the histone marks analyzed. *Sh-Sh2 GFP* (black), Sh1-Sh2 *CHD8*, Sh2-Sh4 *CHD8*, Sh1-Sh4 *CHD8* represent the intersection (number of peaks shared) of two biological replicates, while Sh1-Sh2-Sh4 *CHD8* indicates the intersection of three *CHD8* knock-down replicates (light grey). All intersections of two biological replicates - in any of the possible combinations - show the pattern described in Fig. 1, confirming a decreased number of H3K36me3 peaks following *CHD8* suppression.

B. The bar plot describes the number of total peaks called in control (Sh-*GFP*, dark grey) and *CHD8* knock-down [Sh2-*CHD8* (Talkowski-Gusella), white] ChIP-seq samples for H3K4me1, H3K4me2, H3K36me3. These samples obtained and sequenced at a different time and in a different laboratory [*Talkowski-Gusella laboratory (Massachusetts General Hospital, Boston, MA, United States.)*], confirm a specific decrease in histone H3K36me3 following *CHD8* down-regulation.

Figure Suppl. 3 (related to Fig. 1) CHD8 suppression does not significantly affect enhancer and promoter chromatin regions.

A. B. The bar graphs represent the number of peaks for each histone mark called at strong and weak/poised enhancers a/b genomic regions (A) and active and poised/inactive promoters (B). Grey bars indicate controls (n=2, Sh-*GFP* and Sh-*GFP2*) and white bars refer to *CHD8* knock-down (n=2, Sh2-*CHD8* and Sh4-*CHD8*). Peaks differences between the two conditions at these chromatin states are not statistically significant (two-sided t test, see Materials and Methods for details).

C. E. Metagene profiles display the average of histone H3K27ac (C) and H3K4me1 (E) enrichment (scaled log2 ratio of normalized ChIP value over INPUT control - see also Materials and Methods) in a region of ±2 Kbp upstream the transcriptional start site (TSS) and downstream the transcriptional end site (TES), calculated for control (black line) and *CHD8* knock-down (grey line).

D. F. Plots report Cohen's d effect size statistics of the difference between control and *CHD8* knock-down H3K27ac (D) and H3K4me1 (F) over 2 Kbp around the gene body of protein coding genes. Transcriptional start and end sites are marked by red vertical lines. The effect size along the gene body is negligible for both clusters.

G. H. Volcano plot represent H3K27ac and H3K4me1 peaks differential enrichment. G. All peaks for H3K27Ac are not significant with -log10(FDR) =0, depicted with black dots at the bottom of the plot. The horizontal dashed line represents the threshold for significance at FDR 0.05. H. Significant peaks for H3K4me1 are depicted in black, two on the left side with negative log2(fold change) represent peaks enriched in *CHD8* knock-down, one on the right side with positive log2(fold change) represents a peak enriched in control. Grey dots represent peaks that do not reach the threshold for significance at FDR 0.05, marked by the horizontal dashed line.

Fi*gure Suppl. 4 (related to Fig. 1)* Met*agene profile and heatmaps of H3K36me3 enrichment for affected and not affected genomic locations.*

A. B. Metagene profiles display the average of histone H3K36me3 enrichment (scaled log2 ratio of normalized ChIP value over INPUT control - see also Materials and Methods) of genes directly affected (A) and not affected (B) by *CHD8* suppression in a region of ±2 Kbp upstream the transcriptional start site (TSS) and downstream the transcriptional end site (TES), calculated for control (black line) and *CHD8* knock-down (grey line).

C. Composite heatmaps plot the loci (rows) presenting H3K36me3 enrichment for gene affected (top part) and not affected (bottom part) by *CHD8* suppression in a region of ±2 Kbp upstream the transcriptional start site (TSS) and downstream the transcriptional end site (TES). Genes in heatmaps are ranked based on their library-size normalized ChIP enrichment value relative to INPUT (enrichment score). Blue/red colors indicate high/low histone mark enrichment compared to INPUT.

Figure Suppl. 5 (related to Fig. 2) CHD8 binding correlates with high H3K36me3 and H3K4me3 and elevated RNA expression levels.

A. The heatmaps represent 10 different chromatin states [1. transcriptional initiation, 2. transcriptional elongation, 3. weak transcribe, 4. strong enhancer, 5. weak/poised enhancer a, 6. weak/poised enhancer b, 7. active promoter, 8. inactive/poised promoter, 9. polycomb repressed, 10. heterochromatin/low signal] defined by the combination of different histone marks in control hiNPC as defined by ChromHMM (9). The distribution of CHD8 binding sites (percentage of total CHD8 peaks) across different chromatin states (see Materials and Methods for details) is presented as percentage of the total and color-coded in the heatmap.

B. C. Metagene profiles display the average of histone H3K36me3 (B) and H3K4me3 (C) enrichment (scaled log2 ratio of normalized ChIP value over INPUT control - see also Materials and Methods) in a region of ±2 Kb around the gene body calculated for control hiNPC and for CHD8-bound (#988) and CHD8-unbound genes (#4205). The difference between histone H3K36me3 (B) and H3K4me3 (C) enrichment in CHD8-bound (dark grey) and unbound (light grey) genes is significant. TSS, Transcriptional Start Site; TES, Transcriptional End Site.

D. The box plot shows the average RNA expression level of protein coding genes bound (dark grey) and unbound by CHD8 (light grey). CHD8-bound genes correlate with higher expression levels. TPM: Transcripts Per Kilobase Million. Extreme outliers not displayed. *** = $p < 0.001$, t test statistic.

E. F. Composite heatmaps plot the loci (rows) presenting H3K36me3 (E) and H3K4me3 enrichment (F) for CHD8-bound and CHD8-unbound genes. Genes in heatmaps are ranked based on their library-size normalized ChIP enrichment value relative to INPUT (enrichment score). Blue/red colors indicate high/low histone mark enrichment compared to INPUT.

Figure Suppl. 6 (related to Fig. 2) CHD8-bound genes display a significantly different H3K36me3 enrichment along the gene body between control and CHD8 *knock-down.*

A. Plot reporting Cohen’s d effect size statistics of the difference between control and *CHD8* knock-down H3K36me3 over 2 Kbp around the gene body of protein coding genes for CHD8-bound (blue) and CHD8-unbound genes (red) (transcriptional start and end sites are marked by red vertical lines). The effect size along the gene body is significant for CHD8-bound genes while it remains in the negligible area for CHD8-unbound genes.

B. Plot reporting Cohen’s d effect size statistics of the difference between control and *CHD8* knock-down H3K36me3 over 2 Kbp around the gene body of protein coding genes for Cluster#1 genes. Transcriptional start and end sites are marked by red vertical lines. The effect size along the gene body is significant.

C. D. Plot reporting Cohen’s d effect size statistics of the difference between control and *CHD8* knock-down H3K36me3 over 2 Kbp around the gene body of protein coding genes for Cluster#2 (C.) and Cluster#3 (D.). Transcriptional start and end sites are marked by red vertical lines. The effect size along the gene body is negligible for both clusters.

Figure Suppl. 7 (related to Fig. 2) H3K4me3 and H3K36me3 correlation with RNA expression levels. Histone H3K4me3 enrichment is not affected by CHD8 knock-down.

A. B. Scatter plots report the correlation between H3K36me3 ChIP-seq (A), H3K4me3 ChIP-seq (B) (y-axis, normalized read counts) and RNA-seq (x-axis, normalized read counts) in control hiNPC. Lowess smooth is represented by red lines.

C. Metagene profile showing H3K4me3 enrichment (scaled log2 ratio of normalized ChIP/INPUT) in control (black line) and *CHD8* knock-down (grey line) on the TSS and along the gene body of protein coding genes (TSS, Transcriptional Start Site; TES, Transcriptional End Site).

D. Composite heatmaps plot the loci (rows) presenting H3K4me3 enrichment for protein coding genes in control (left) and *CHD8* knock-down (right). A region spanning ± 2Kb around the gene body is analyzed. Genes in heatmaps are ranked based on their library-size normalized ChIP enrichment value relative to INPUT (enrichment score). Blue/red colors indicate high/low histone H3K36me3 enrichment compared to INPUT.

E. Plot reporting Cohen's d effect size statistics of the difference between control and *CHD8* knock-down H3K4me3 over 2 Kbp around the gene body of protein coding genes (transcriptional start and end sites are marked by red vertical lines). The effect size of the difference between H3K4me3 enrichment in control and *CHD8* knock-down along the whole region remains in the negligible area.

Figure Suppl. 8 (related to Fig. 2) GO terms enrichment analysis for genes presenting lower H3K36me3 upon CHD8 *suppression.*

Bar plot presenting the Biological Process GO terms significantly enriched in genes belonging to Cluster #3, with low-negligible CHD8 binding enrichment in control hiNPCs and a lower H3K36me3 enrichment in *CHD8* knock-down (Fig. 2 E). Bars are colored according to -log10 (adjusted p values) and x-axis represents the number of genes per term.

Figure Suppl. 9 (related to Fig. 3) CHD8-suppression elicited reduction in H3K36me3 doesn't correlate with altered transcription, but with significant changes in RNA alternative splicing – validation by rMATS, independent approach.

A. B. The box plots show the RNA expression level average for protein coding genes (A) and for genes that lose H3K36me3 peaks following *CHD8* knock-down (B). Control condition is shown in dark grey, *CHD8* knock-down in white; genes bound (left) and unbound by CHD8 (right) are also indicated. TPM: Transcripts Per Kilobase Million. Extreme outliers not shown.

C. The scatter plot reports the H3K36me3 peaks enrichment log2(fold change) vs gene expression log2(fold change). Red horizontal dashed lines represent enrichment fold change thresholds for significance (1, -1). For some genes, multiple peaks with log2(fold change) values can be present.

D. E. Venn diagrams represent the overlap between genes losing H3K36me3 peaks following *CHD8* knock-down (losing H3K36me3 in *CHD8* KD) and genes presenting altered alternative splicing events as detected by rMATS (AS rMATS) (D), and the overlap between genes bound by CHD8 (CHD8-bound) and genes presenting altered alternative splicing events as detected by rMATS (AS rMATS) (E). Number of genes for each condition is indicated. The enrichment significance for each intersection is measured by Fisher's exact test and represented by colors. Color coded legend: -log10 (p value).

F. Stacked bar plot represents the 1484 differential alternative splicing events detected by rMATS, distributed by event type. SE, skipped event; RI, retained intron; MX, mixed event; A3, alternative 3'; A5, alternative 5'.

G. The scatter plot of H3K36me3 enrichment [log2(fold change)] vs alternative splicing (dPSI) is presented. H3K36me3 peaks (DiffBind) and exons (SUPPA) undergoing alternative splicing events were intersected by coordinates (min overlap 1bp). Red horizontal dashed lines represent enrichment fold change thresholds for significance (1, -1). Blue vertical dashed lines represent dPSI thresholds for significance (-0.20, 0.20).

Figure Suppl. 10 (related to Fig. 3) CHD8-suppression elicited reduction in H3K36me3 correlates with significant alterations in RNA alternative splicing in mouse ASD models.

A. B. Stacked bar plot represents the 1586 and 1615 differential alternative splicing events detected by SUPPA in P5 cortex (10)(A) and mNPC (11)(B) samples respectively. Event type is indicated as well as positive and negative deltaPSI. SE, skipped event; RI, retained intron; MX, mixed event; A3, alternative 3'; A5, alternative 5'.

C. Venn diagram represents the overlap between genes presenting altered alternative splicing events as detected by SUPPA in iPS-derived NPC presenting reduced levels of *CHD8* (Sh) vs P5 cortex vs mNPC.

D. E. F. Venn diagrams represent the overlap between genes presenting altered alternative splicing events as detected by SUPPA in iPS-derived NPC presenting reduced levels of *CHD8* (Sh) vs P5 cortex (D), Sh vs mNPC (E) and mNPC vs P5 cortex (F). In C. D. E. and F. Number of genes for each condition is indicated. The enrichment significance for each intersection is measured by Fisher's exact test and represented by colors. Color coded legend: -log10 (p value).

G. H. I. Bar plots presenting the top 20 Biological Process GO terms significantly enriched in genes presenting alternative splicing in iPS-derived NPC presenting reduced levels of *CHD8* (Sh) (G), P5 cortex (H) and mNPC (I). Bars are colored according to -log10 (adjusted p values) and x-axis represents the number of genes per term.

Figure Suppl. 11 (related to Fig. 4) CHD8 interactome from nuclear lysate of iPS-derived NPC and murine embryonic stem cells (ESC).

A. Representative western blot images report molecular characterization of nuclear (left columns) and cytoplasmic (right columns) fractions from hNPC. Poly [ADP-ribose] polymerase 1 (PARP1), histone H3 (H3), LaminA/C were used to identify nuclear compartment; HSP90 and GAPDH were used as cytosolic markers.

B. Venn diagrams represent the overlap between statistically significant proteins identified by pulling down CHD8 with Novus Biologicals NB-10060417 antibody (IP 17), in three biological replicates (single replicates IP 17A/B/C analyzed separated)

C. Venn diagrams represent the overlap of statistically significant proteins identified by pulling down CHD8 with Novus Biologicals NB-10060418 antibody (IP 18), in three independent experiments (single replicates IP A/B/C and C analyzed separated). Number of proteins for each condition is indicated. List of proteins present in Suppl. Table 1.

D. The bar plot represents KEGG, GO biological process and GO molecular function terms significantly enriched for proteins in the overlap presented in D and listed in E. The bars are ordered according to adjusted p values in -log10 scale, the x-axis represents the number of proteins enriched for each term. The threshold for significance is set at adjusted p value 0.05 for KEGG and GO molecular function terms while top 9 GO biological process terms are shown in figure. Full list of KEGG and GO terms are reported in Suppl. Table 1.

E. Venn diagrams represent the overlap between statistically significant proteins identified in MS with NB-10060417 antibody in hNPC (IP 17A-B averaged and analyzed together) and equivalent MS experiments, pulling down CHD8 with Bethyl A301-224A antibody in mESC. Number of proteins for each condition is indicated. List of proteins present in Suppl. Table 1.

F. Bar plot represents KEGG, GO biological process and GO molecular function terms significantly enriched in proteins from the intersection in D. The bars are ordered according to adjusted p values in -log10 scale, the x-axis represents the number of proteins enriched for each term. The threshold for significance is set at adjusted p value 0.05 for KEGG while top 9 GO biological process and GO molecular function terms are shown in figure. Complete list of KEGG and GO terms reported inList of proteins present in Suppl. Table 1.

G. Representative western blot images depict immunoprecipitation by endogenous, full-length CHD8 in nuclear extracts by two different antibodies CHD8 NB100-60417 (IP CHD8 17) and NB100-60418 (IP CHD8 18). A strong reproducible enrichment compared to Input (Input, 15 μg of nuclear lysate) and Rabbit IgG control (IgG) is evident. The interaction between endogenous CHD8 and hnRNPL in hNPC is not dependent on the presence of Ethidium Bromide (EtBr) or DNAse I treatment. NT, no treatment. CHD8 high exp., high exposure = 30 sec; CHD8 low exp, low exposure = 10 sec. HnRNPL high exp., high exposure = 60 sec; hnRNPL low exp, low exposure = 4 sec.

H. The bar plot depicts the quantification of the WB bands (hnRNPL/CHD8) comparing the different treatment conditions (Ethidium Bromide (EtBr), DNAse I (DNAseI), RNAse A (RNAseA), no treatment (NT)). The WB bands were quantified using Image-J software, mean +/- standard deviation of biological replicates is presented. Student t-test for one mean population performed with NS $P>0.05$, * $P\leq0.05$.

Suppl. Fig. 12 (related to Fig. 5). siRNA-mediated knock-down of hnRNPL in iPS-derived NPC.

A. Representative western blot images report molecular characterization of hnRNPL from hNPC treated with 200 pM siRNAs for 48 and 72h post electroporation. 3 different 27-mer siRNAs (si-A, si-B, si-C) against *hnRNPL* were used together with si-scambled (si-Scr) control. HSP90 was used as loading control.

B. The bars represent the quantification of the western blot images reporting hnRNPL protein intensity relative to si-Scr control at 48 and 72h post electroporation.

C. The scatter plot describes the Principal Component Analysis (PCA) of the RNAseq data obtained by hiNPC cells treated with si-C against *hnRNPL* versus si-Scr control. 4 different biological replicates for each experimental condition were analyzed. PC1 (x-axis, 54% variance) and PC2 (y-axis, 24% variance) components are presented.

D. The bar plot reports the expression levels (Transcript Per Million, TPM) of the members of the hnRNPs family. Si-C against *hnRNPL* and si-Scr are compared. 4 Different replicates for experimental condition are presented.

E. Stacked bar plot represents the differential alternative splicing events detected by SUPPA, distributed by event type sof RNAseq data from *hnRNPL* knock-down in HepG2 and K562 cells. SE, skipped event; RI, retained intron; MX, mixed event; A3, alternative 3'; A5, alternative 5'; AF, alternative first exon; AL, alternative last exon.

Supplementary references

1. Sugathan, A., Biagioli, M., Golzio, C., Erdin, S., Blumenthal, I., Manavalan, P., Ragavendran, A., Brand, H., Lucente, D., Miles, J. *et al.* (2014) CHD8 regulates neurodevelopmental pathways associated with autism spectrum disorder in neural progenitors. *Proc Natl Acad Sci U S A*, **111**, E4468-4477.
2. Ramirez, F., Ryan, D.P., Gruning, B., Bhardwaj, V., Kilpert, F., Richter, A.S., Heyne, S., Dundar, F. and Manke, T. (2016) deepTools2: a next generation web server for deep-sequencing data analysis. *Nucleic acids research*, **44**, W160-165.
3. Davis, C.A., Hitz, B.C., Sloan, C.A., Chan, E.T., Davidson, J.M., Gabdank, I., Hilton, J.A., Jain, K., Baymuradov, U.K., Narayanan, A.K. *et al.* (2018) The Encyclopedia of DNA elements (ENCODE): data portal update. *Nucleic acids research*, **46**, D794-D801.
4. Consortium, E.P. (2012) An integrated encyclopedia of DNA elements in the human genome. *Nature*, **489**, 57-74.
5. Quinlan, A.R. and Hall, I.M. (2010) BEDTools: a flexible suite of utilities for comparing genomic features. *Bioinformatics*, **26**, 841-842.
6. Marini, C., Porro, A., Rastetter, A., Dalle, C., Rivolta, I., Bauer, D., Oegema, R., Nava, C., Parrini, E., Mei, D. *et al.* (2018) HCN1 mutation spectrum: from neonatal epileptic encephalopathy to benign generalized epilepsy and beyond. *Brain : a journal of neurology*, **141**, 3160-3178.
7. Nava, C., Dalle, C., Rastetter, A., Striano, P., de Kovel, C.G., Nabbout, R., Cances, C., Ville, D., Brilstra, E.H., Gobbi, G. *et al.* (2014) De novo mutations in HCN1 cause early infantile epileptic encephalopathy. *Nature genetics*, **46**, 640-645.

8. Luk, I.S., Shrestha, R., Xue, H., Wang, Y., Zhang, F., Lin, D., Haegert, A., Wu, R., Dong, X., Collins, C.C. *et al.* (2017) BIRC6 Targeting as Potential Therapy for Advanced, Enzalutamide-Resistant Prostate Cancer. *Clinical cancer research : an official journal of the American Association for Cancer Research*, **23**, 1542-1551.

9. Ernst, J. and Kellis, M. (2012) ChromHMM: automating chromatin-state discovery and characterization. *Nature methods*, **9**, 215-216.

10. Suetterlin, P., Hurley, S., Mohan, C., Riegman, K.L.H., Pagani, M., Caruso, A., Ellegood, J., Galbusera, A., Crespo-Enriquez, I., Michetti, C. *et al.* (2018) Altered Neocortical Gene Expression, Brain Overgrowth and Functional Over-Connectivity in Chd8 Haploinsufficient Mice. *Cerebral cortex*, **28**, 2192-2206.

11. Sood, S., Weber, C.M., Hodges, H.C., Krokhotin, A., Shalizi, A. and Crabtree, G.R. (2020) CHD8 dosage regulates transcription in pluripotency and early murine neural differentiation. *Proceedings of the National Academy of Sciences of the United States of America*, **117**, 22331-22340.

Kerschbamer_SupFig1

A

Name	Transcript ID	Sh-*GFP1* (TPM)	Sh-*GFP2* (TPM)	Sh4-*CHD8* (TPM)	Sh2-*CHD8* (TPM)	Sh1-*CHD8* (TPM)
CHD8_201	ENST00000430710.7	17.34	17.40	7.45	7.42	9.14
CHD8_212	ENST00000557364.5	13.80	10.76	5.95	4.67	6.44

B

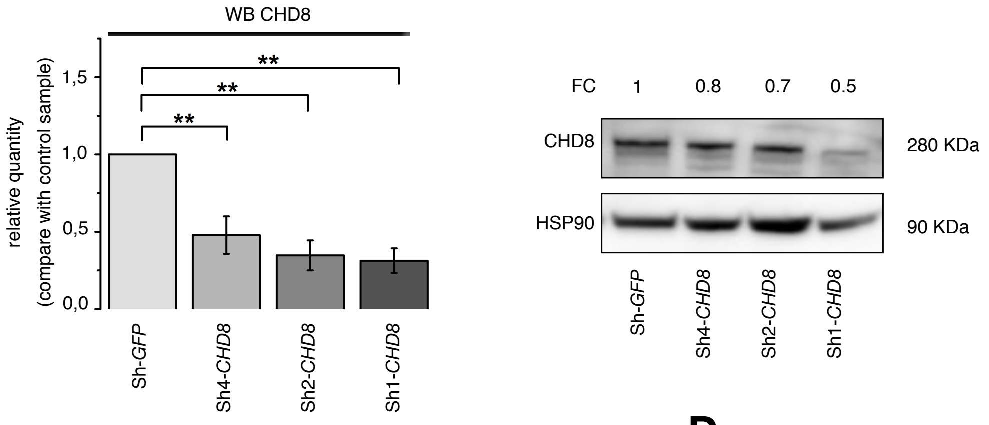

C

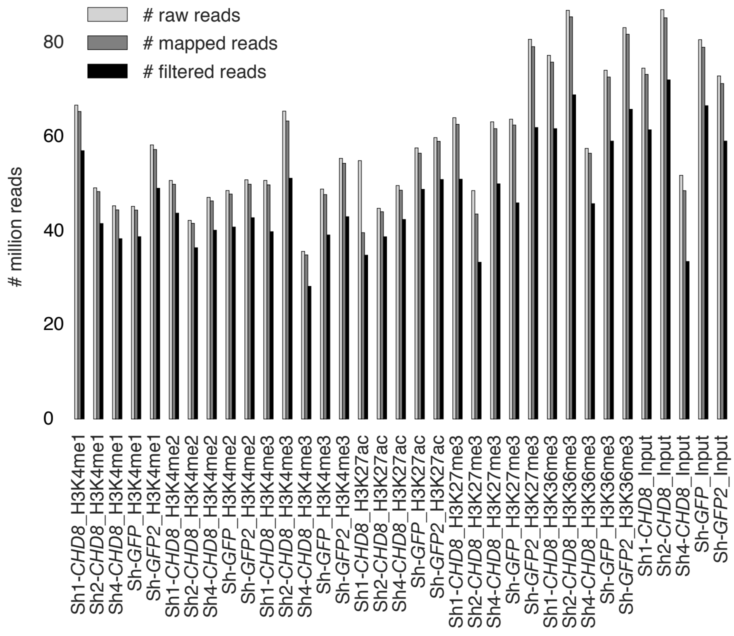

D

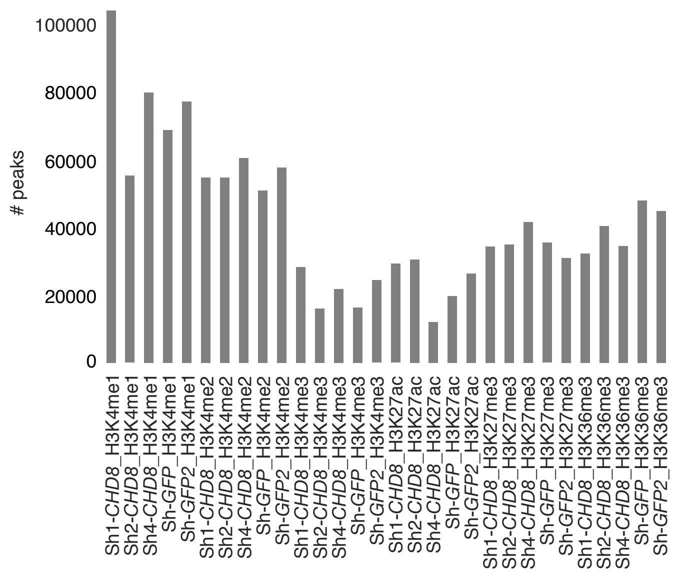

E

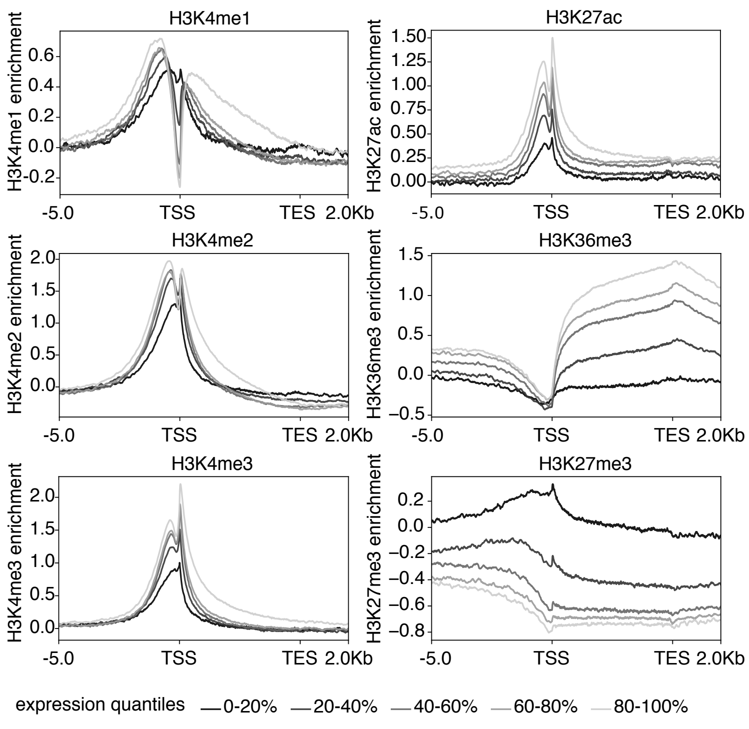

F

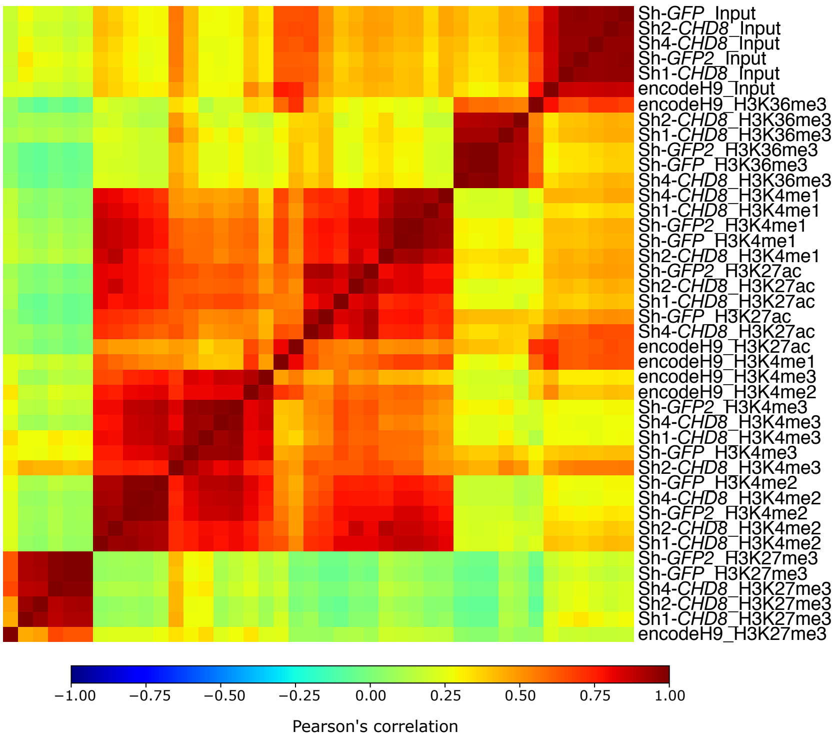

Kerschbamer_SupFig2

A

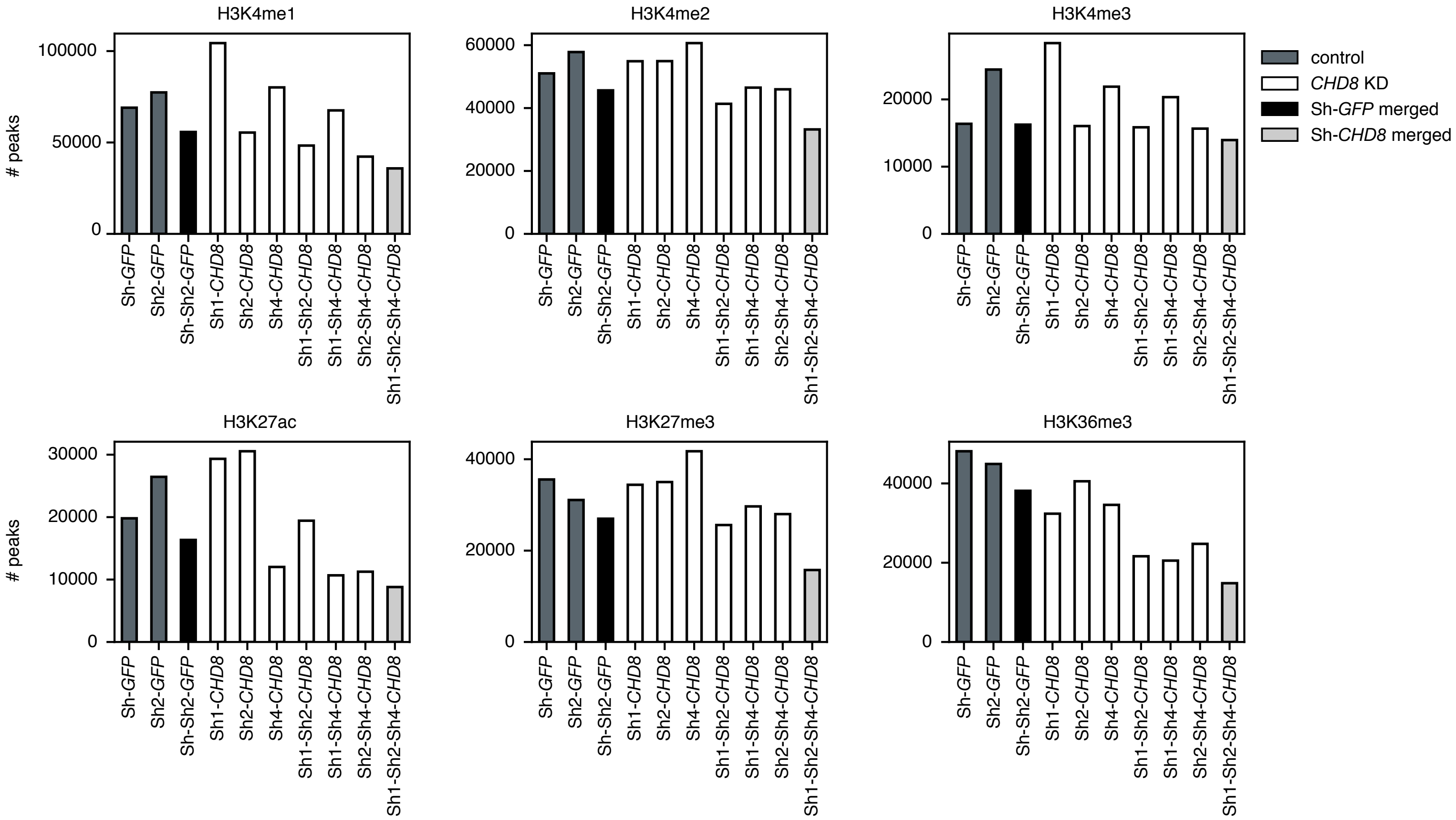

B

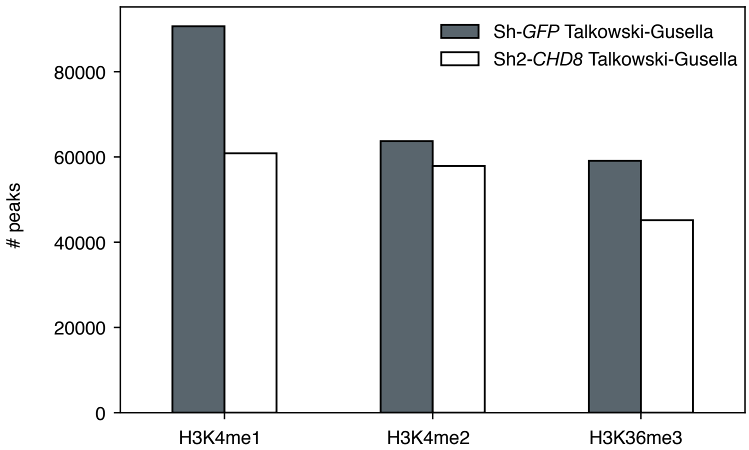

Kerschbamer_SupFig3

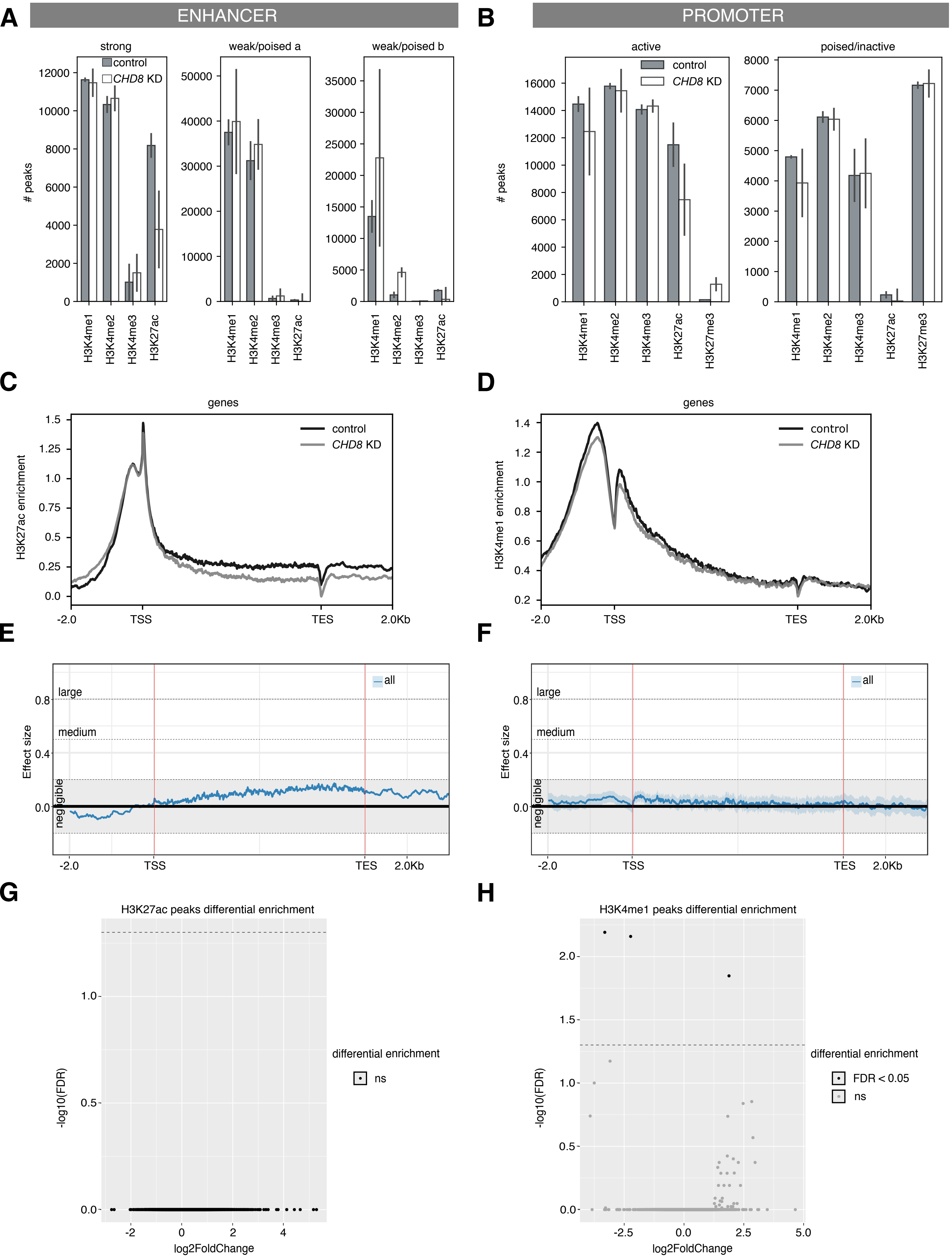

Kerschbamer_SupFig4

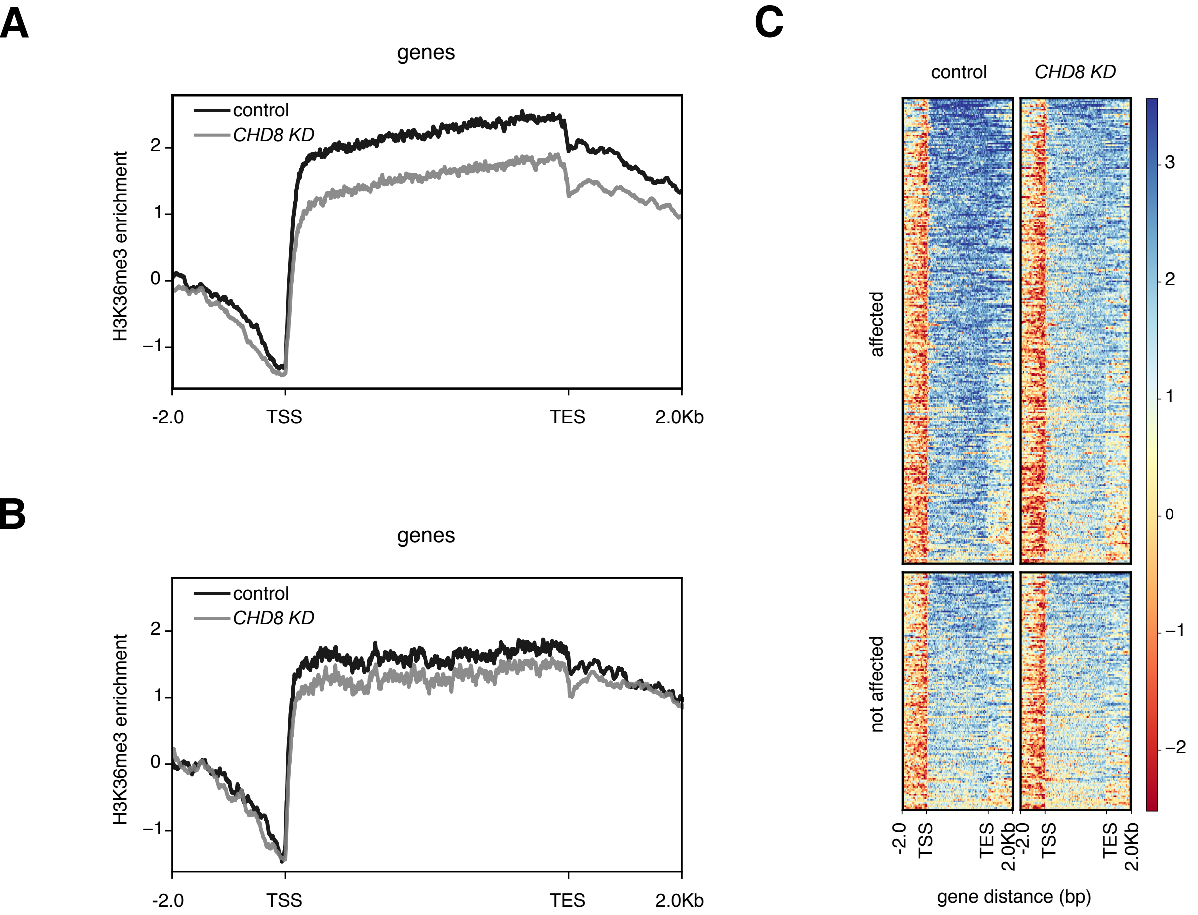

Kerschbamer_SupFig5

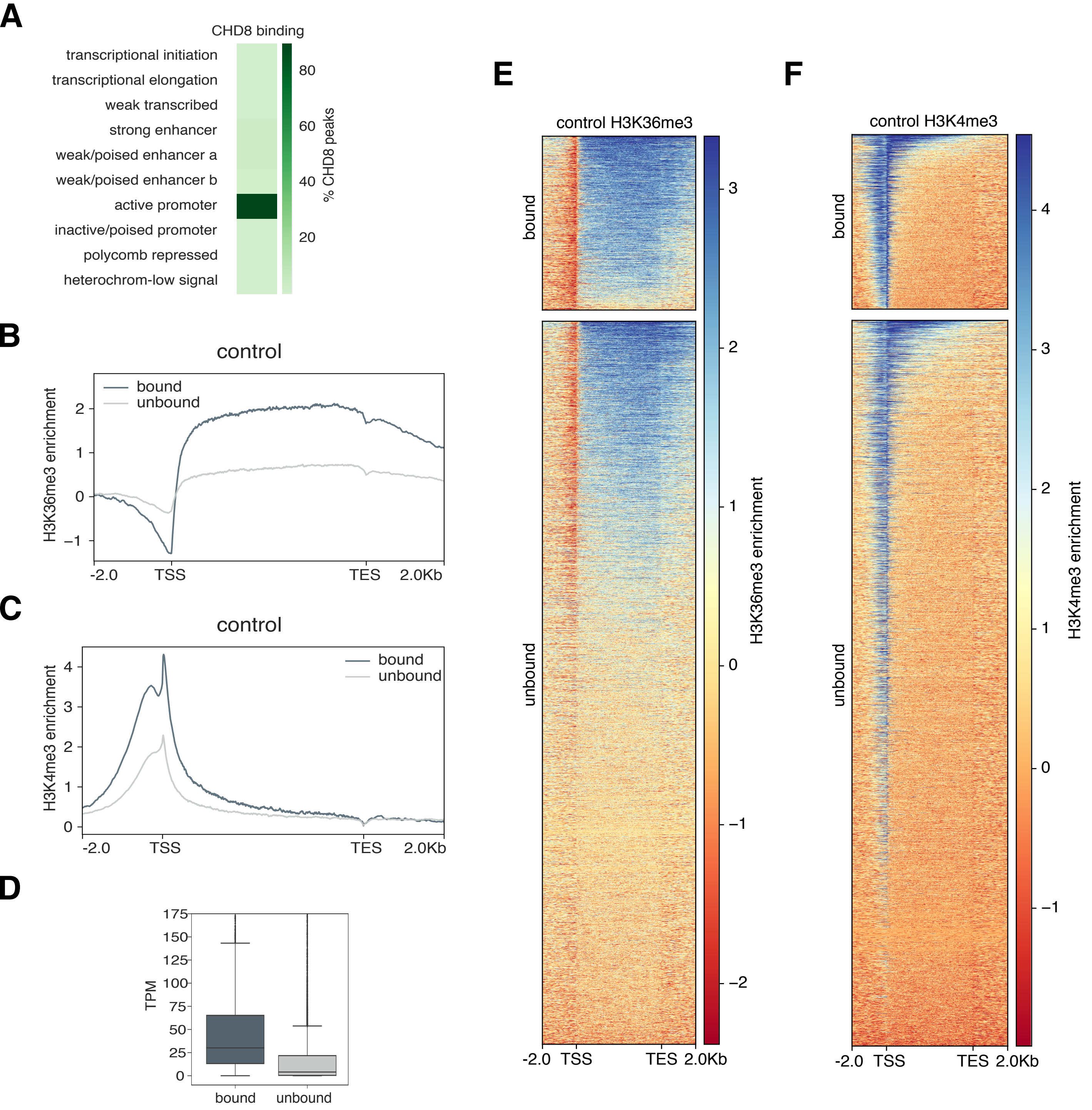

Kerschbamer_SupFig6

A

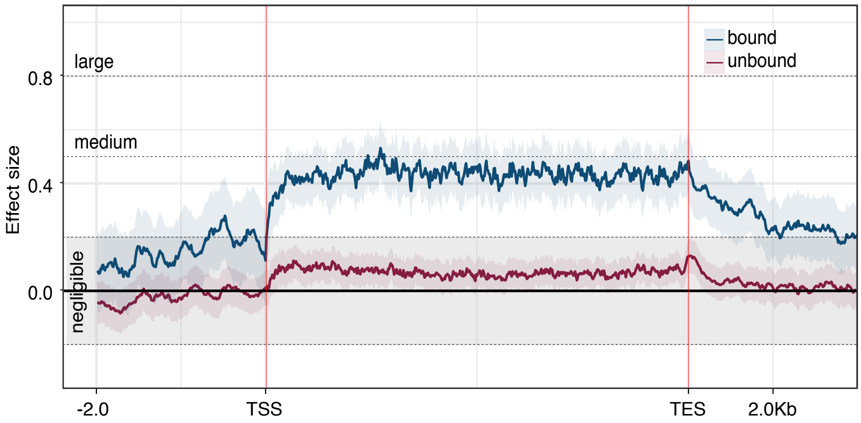

B

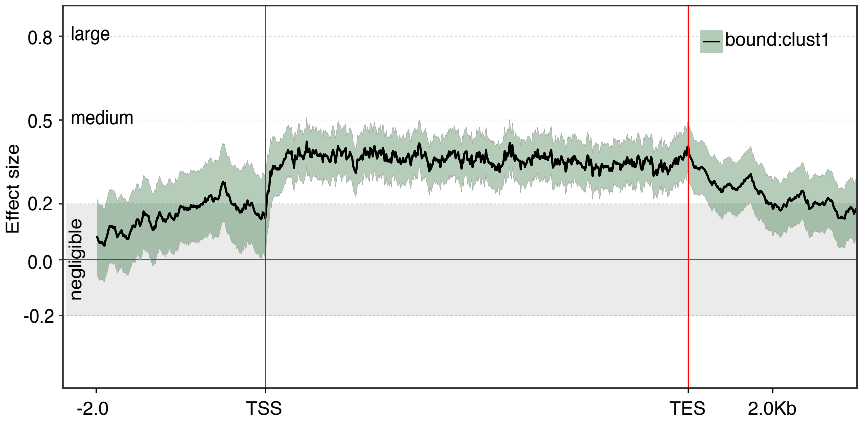

C

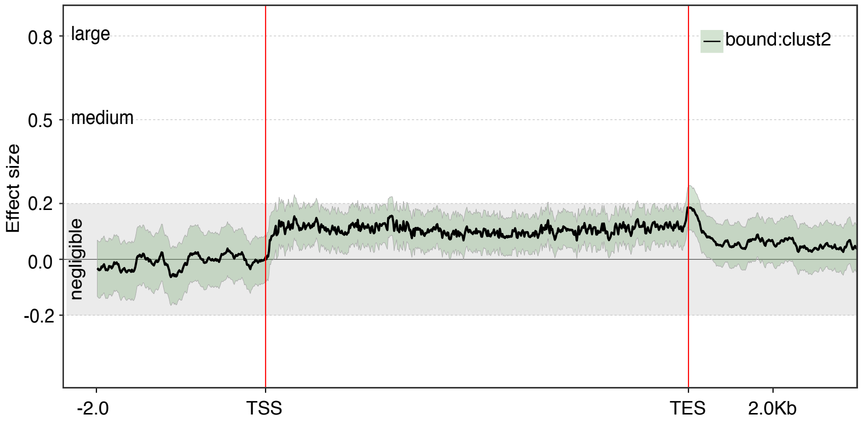

D

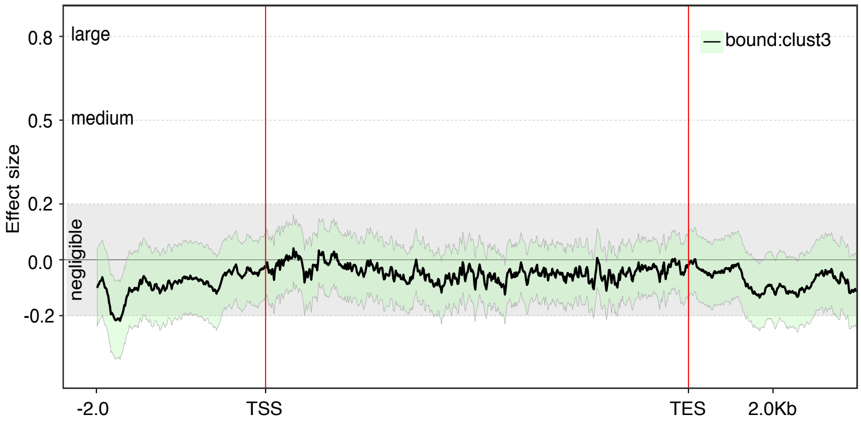

Kerschbamer_SupFig7

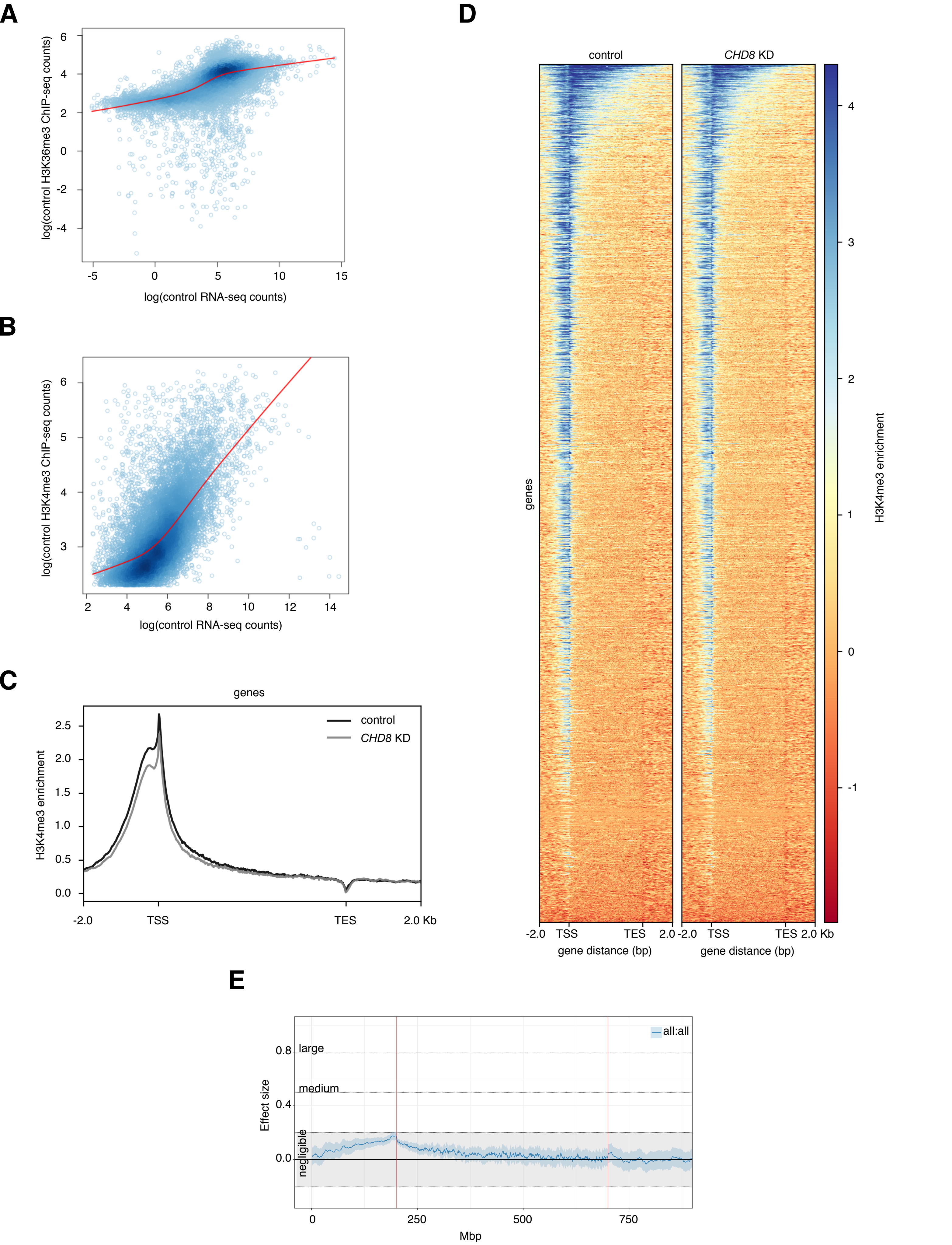

Kerschbamer_SupFig8

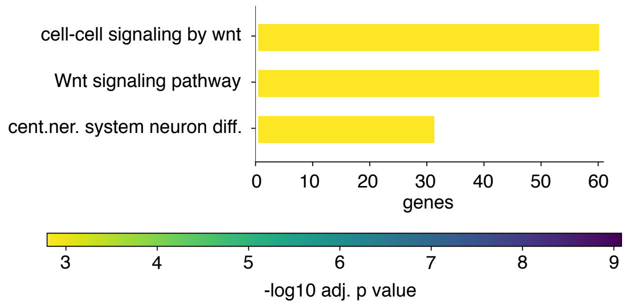

Kerschbamer_SupFig9

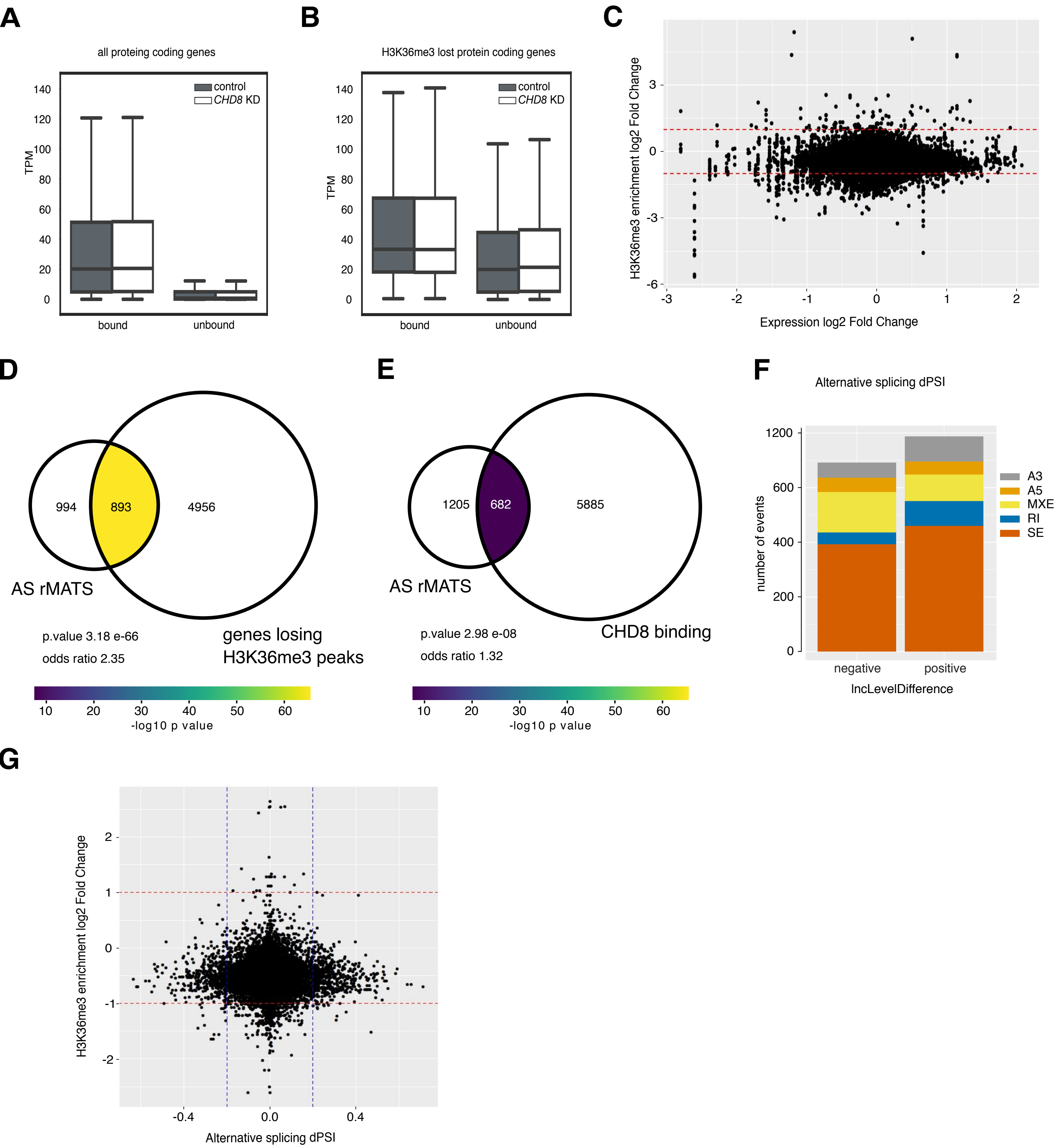

Kerschbamer_SupFig10

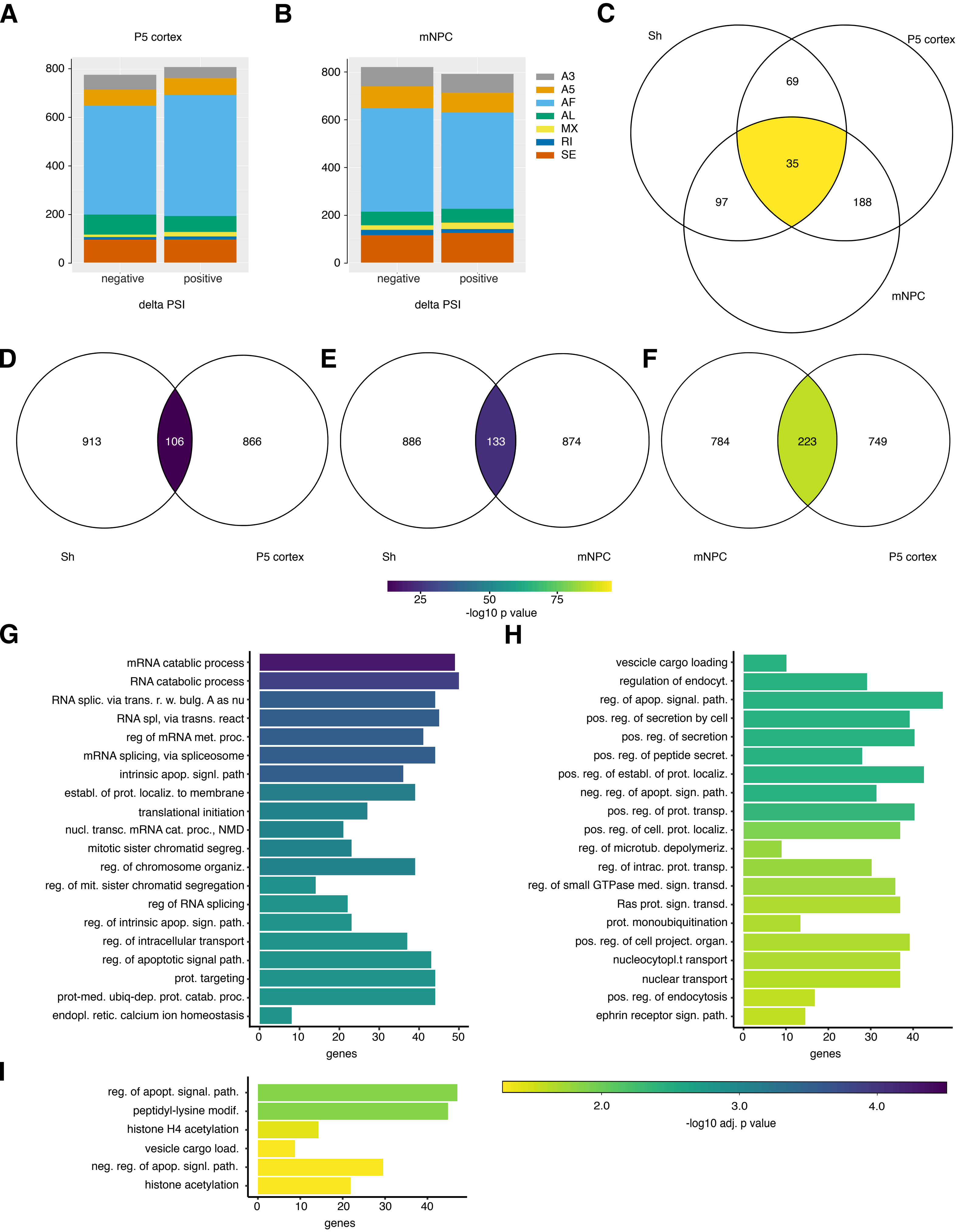

Kerschbamer_SupFig11

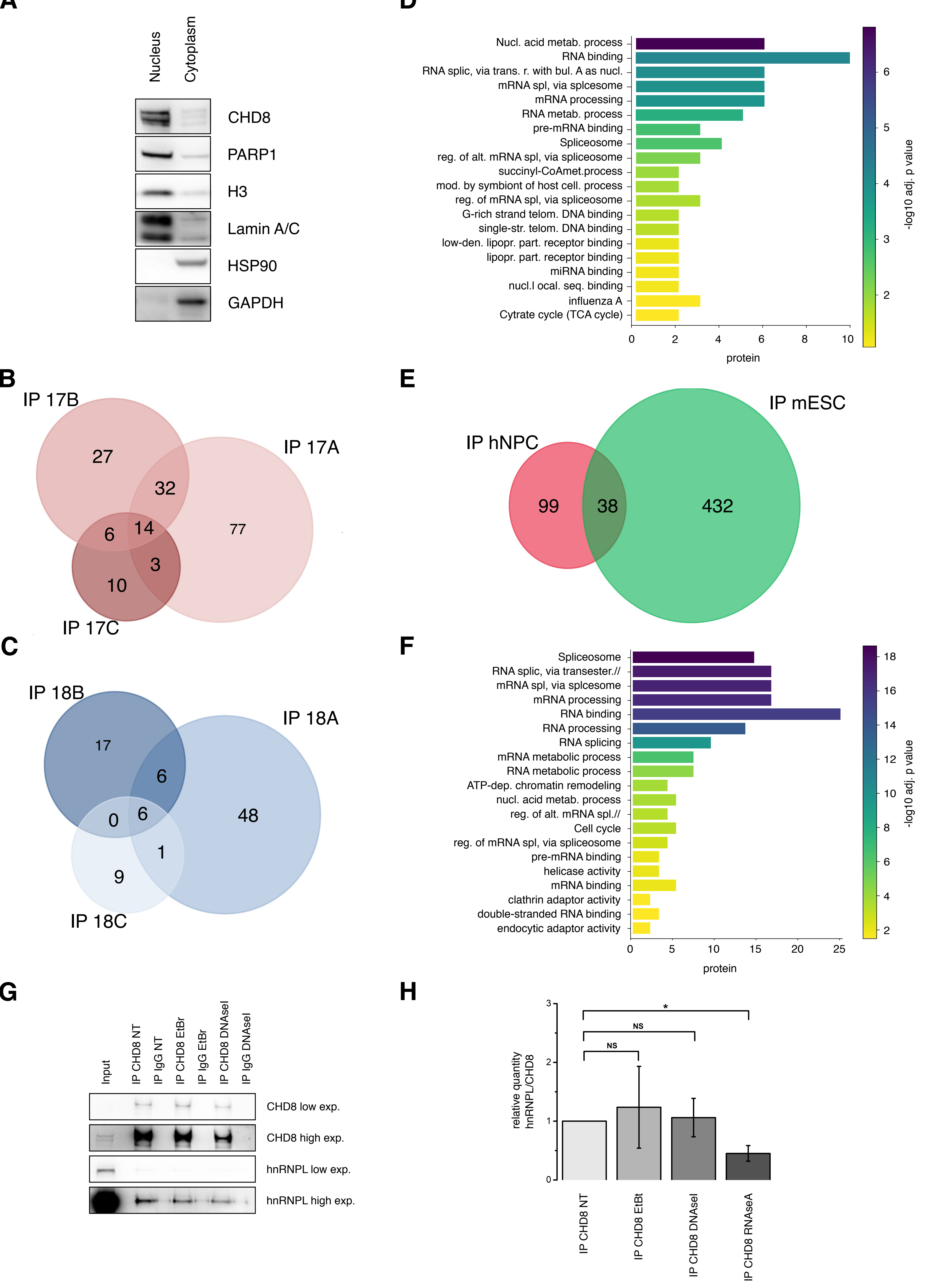

Kerschbamer_SupFig12

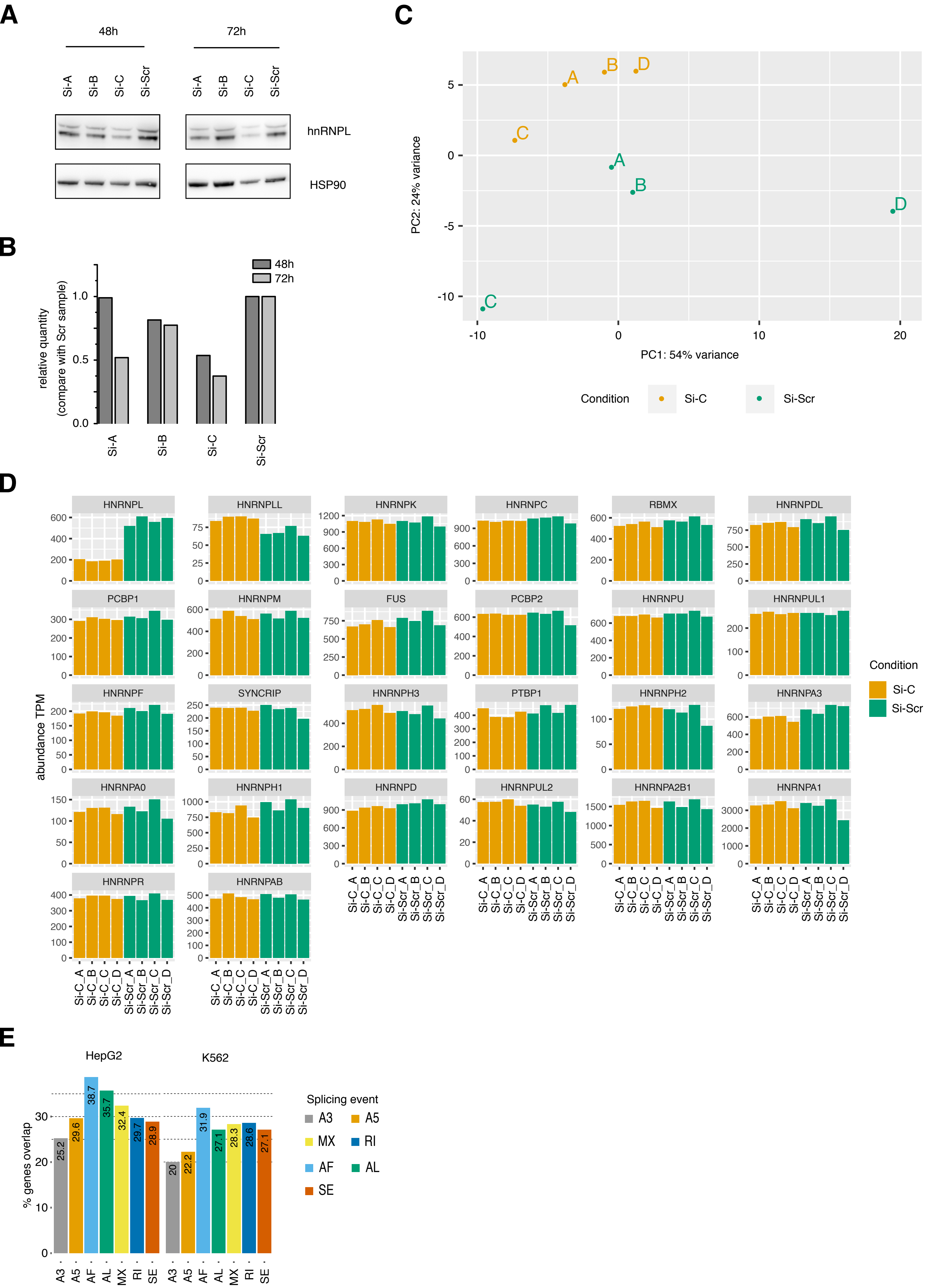

Supplement: gkac1134_Supplemental_Files [file gkac1134_supplemental_files.zip › Supplementary_Kerschbamer_Oct 2022.pdf]
